# The role of cardiac resident macrophage in cardiac aging

**DOI:** 10.1111/acel.14008

**Published:** 2023-10-10

**Authors:** Jiayu Li, Yanguo Xin, Zhaojia Wang, Jingye Li, Weiping Li, Hongwei Li

**Affiliations:** ^1^ Department of Cardiology, Cardiovascular Center, Beijing Friendship Hospital Capital Medical University Beijing China; ^2^ Laboratory for Clinical Medicine Beijing Friendship Hospital, Capital Medical University Beijing China; ^3^ Beijing Key Laboratory of Metabolic Disorder Related Cardiovascular Disease Beijing China

**Keywords:** aging, heart, immunity, macrophage

## Abstract

Advancements in longevity research have provided insights into the impact of cardiac aging on the structural and functional aspects of the heart. Notable changes include the gradual remodeling of the myocardium, the occurrence of left ventricular hypertrophy, and the decline in both systolic and diastolic functions. Macrophages, a type of immune cell, play a pivotal role in innate immunity by serving as vigilant agents against pathogens, facilitating wound healing, and orchestrating the development of targeted acquired immune responses. Distinct subsets of macrophages are present within the cardiac tissue and demonstrate varied functions in response to myocardial injury. The differentiation of cardiac macrophages according to their developmental origin has proven to be a valuable strategy in identifying reparative macrophage populations, which originate from embryonic cells and reside within the tissue, as well as inflammatory macrophages, which are derived from monocytes and recruited to the heart. These subsets of macrophages possess unique characteristics and perform distinct functions. This review aims to summarize the current understanding of the roles and phenotypes of cardiac macrophages in various conditions, including the steady state, aging, and other pathological conditions. Additionally, it will highlight areas that require further investigation to expand our knowledge in this field.

## Graphical abstract

Advancements in modern medicine have significantly increased lifespan, as we age, the functionality of many of our organ systems begins to decline, such as the heart. The differentiation of cardiac macrophages (CCR2− and CCR2+ populations) recruited to the heart, maintaining the physiological processes of heart including neonatal regeneration and adoptive remodeling. During the aging process, specific macrophage subpopulation possess unique characteristics and perform distinct functions.

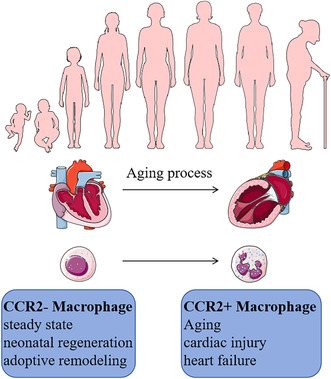

**Abbreviations**
AGEs: advanced glycation end products
ATP: adenosine triphosphate
BMDMs: bone marrow‐derived macrophages
BMMSCs: bone marrow‐derived mesenchymal stem cells
CCR2: chemokine receptor‐2
CRMs: cardiac tissue‐resident macrophages
CSF‐1: colony‐stimulating factor
CVD: cardiovascular disease
DAMPs: damage‐associated molecular patterns
EAT: epicardial adipose tissue
ECM: extracellular matrix
FAO: fatty acid oxidation
GAPDH: glyceraldehyde‐phosphate dehydrogenase
HFpEF: heart failure with preserved ejection fraction
HIF‐1α: hypoxia‐inducible factor 1 alpha
IGF‐1: insulin‐like growth factor 1
LYVE1: lymphatic vessel endothelial hyaluronan receptor 1
MertK: Mer proto‐oncogene tyrosine kinase
MHC‐II: major histocompatibility complex
MI: myocardial infarction
MMPs: matrix metalloproteinases
NADPH: Nicotinamide adenine dinucleotide phosphate
NK cells: natural killer cells
OXPHOS: oxidative phosphorylation
PDGF: platelet derived growth factor
ROS: reactive oxygen species
TGF: transforming growth factor
TIMD4: T cell immunoglobulin and mucin domain‐containing 4
TLRs: toll‐like receptors
TNF‐α: tumor necrosis factor α
TRPV4: transient receptor potential cation channel subfamily V member 4
VEGF: vascular endothelial growth factor

## 1 INTRODUCTION

Based on data provided by the Chinese government, the demographic group comprising individuals aged 65 and above has attained a proportion of 11.9%, signifying a 0.5% rise compared to the preceding year. Scholars anticipate that advancements in health care and enhancements in living standards may lead to a potential increase of 4 years in life expectancy for the global population by the year 2040. Notably, certain nations, including Japan, Singapore, Spain, and Switzerland, exhibit an average life expectancy surpassing 85 years. These projections underscore the substantial potential for notable advancements in life expectancy and the consequent aging of populations in the forthcoming decades (Foreman et al., 2018). The aging of the population plays a substantial role in the heightened occurrence of age‐related ailments, specifically CVD (Donato et al., 2018). By the year 2030, approximately 20% of the American population will surpass the age of 65, and nearly half of them will be afflicted with cardiovascular disease (Benjamin et al., 2017). Globally, CVD persists as the primary cause of mortality, with cardiac aging recognized as a significant autonomous risk element (Ren & Zhang, 2018). Consequently, it becomes imperative to explore the underlying mechanisms of cardiac aging and discover strategies to alleviate or avert its progression.

The investigation into the involvement of the immune system in maintaining cardiac equilibrium, the aging process, and pathological conditions is an expanding area of study. The cardiac structure encompasses a diverse range of cell types, namely cardiomyocytes, fibroblasts, endothelial cells, smooth muscle cells, pericytes, endocardial cells, epicardial cells, as well as various immune cells including monocytes, macrophages, dendritic cells, T cells, natural killer (NK) cells, mast cells, and B cells (Koenig et al., 2022; Pinto et al., 2016; Tucker et al., 2020). Among these immune populations, macrophages are the predominant cellular component. The immune landscape within the heart is dynamic, exhibiting significant shifts in composition and phenotype based on the specific disease context (e.g., infection, sterile injury, hemodynamic changes, aging, myocarditis). In this review, we will focus on discussing the inflammatory response in the heart following acute myocardial injury.

Through the examination of the immune response in cardiac injury, valuable insights can be obtained regarding the underlying mechanisms of inflammation, thereby potentially enabling the identification of therapeutic targets to mitigate the adverse consequences of acute myocardial injury. It is imperative to comprehend the dynamic behavior of macrophages and their interactions with other immune and stromal cells within the cardiac context, as this knowledge is pivotal for elucidating the mechanisms that drive cardiac inflammation and pathology. By unraveling the intricate immune responses, the identification of novel therapeutic targets and the development of interventions to modulate the immune landscape become feasible prospects.

## 2 CARDIAC‐RESIDENT MACROPHAGES

The immune milieu within the cardiac tissue experiences dynamic alterations as it progresses from a state of equilibrium and vigilance to an inflammatory condition. Among the immune cells present, tissue‐resident macrophages prevail as the predominant cellular entity (Ramos et al., 2017). As constituents of the innate immune system, macrophages possess specialized phagocytic abilities that enable them to identify and eradicate apoptotic cells and pathogens. Additionally, they assume a pivotal role in intercellular communication with neighboring stromal and immune cells.

The traditional belief that macrophages originate exclusively from bone marrow monocytes and spleen macrophages has been the subject of controversy (van Furth & Cohn, 1968). Recent studies have presented evidence indicating that tissue‐resident macrophages, including those found in various organs such as the brain, spleen, liver, lung, bone marrow, kidney, pancreas, peritoneum, and heart, are established during prenatal development, persist throughout an individual's lifespan, and possess the ability to self‐renew locally (Ginhoux & Jung, 2014; Nahrendorf & Swirski, 2016). In the steady state, resident macrophages in the heart make up approximately 5%–10% of nonmyocytes (Heidt et al., 2014; Pinto et al., 2016). The spindle‐shaped cardiac macrophages exhibit close interactions with myocytes, endothelial cells, and fibroblasts. Research utilizing genetic fate mapping and lineage‐tracing techniques has revealed that the majority of these resident cardiac macrophages derive from embryonic yolk sacs and fetal livers (Epelman et al., 2014). Furthermore, these resident macrophages maintain their population by undergoing proliferation approximately once per month (Epelman et al., 2014; Heidt et al., 2014).

**FIGURE 1:**
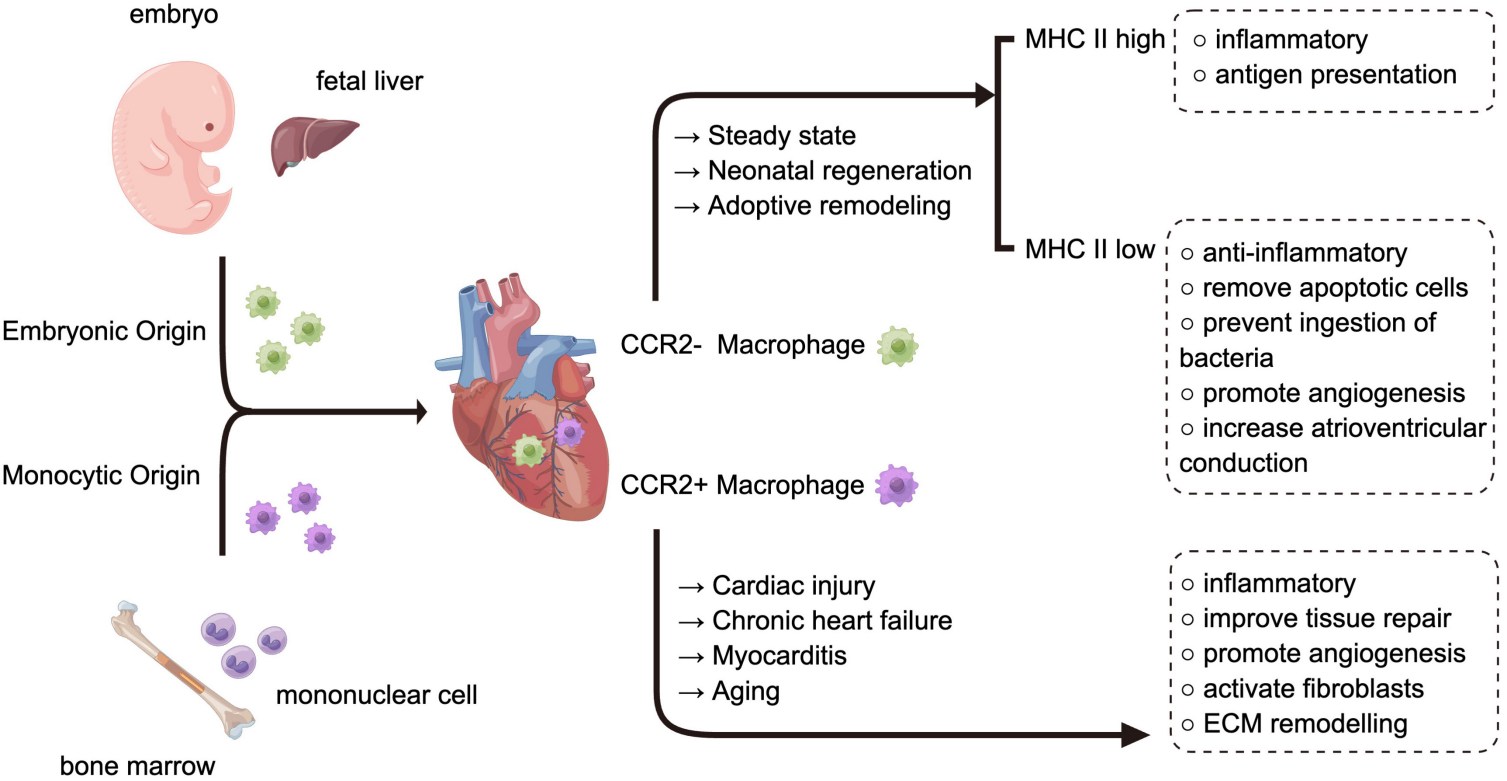
The distribution and prominent characteristics of different subsets of macrophages in the heart are of academic interest. In the steady state, during neonatal heart regeneration, and in adoptive remodeling, the heart predominantly harbors tissue‐resident macrophages lacking the chemokine receptor‐2 (CCR2−). However, in cases of sterile injury, myocarditis, chronic heart failure, and aging, there is a notable transition toward an expansion of CCR2+ macrophages.

Various methods, including the use of cell surface markers such as chemokine receptor‐2 (CCR2), lymphatic vessel endothelial hyaluronan receptor 1 (LYVE1), and T cell immunoglobulin and mucin domain‐containing 4 (TIMD4), genetic fate mapping, transcriptomic profiling, and functional analysis, have been employed to identify and classify tissue‐resident cardiac macrophages (Bajpai et al., 2019; Chakarov et al., 2019; Dick et al., 2019; Epelman et al., 2014; Lavine et al., 2014; Leid et al., 2016). In the steady state, the predominant immune cells in the heart are characterized as CCR2‐ LYVE1+TIMD4+ tissue‐resident macrophages. These long‐lived macrophages originate from hematopoietic progenitors that develop in the yolk sac during embryonic development and eventually seed the heart, during embryonic development (Bajpai et al., 2019; Gomez Perdiguero et al., 2015; Lavine et al., 2014; Leid et al., 2016; Nahrendorf et al., 2007). Cardiac tissue‐resident macrophages (CRMs) are sustained independently of monocyte influx through local proliferation within the heart (Figure 1).

The elucidation of tissue‐resident cardiac macrophages' origins, characteristics, and maintenance offers significant contributions to our comprehension. By delineating the heterogeneity of subpopulations within cardiac macrophages, we can delve deeper into their unique roles and functions concerning cardiac homeostasis, aging, and disease.

Several studies have elucidated the distinct and tissue‐specific roles of CRMs, similar to other macrophage populations residing in tissues such as microglia, Kupffer cells, and alveolar macrophages (Ginhoux et al., 2010; Gomez Perdiguero et al., 2015; Guilliams et al., 2013; Yona et al., 2013). Throughout development and maintenance of equilibrium, CRMs actively participate in diverse processes, including the removal of apoptotic cells, stimulation of coronary growth and organization, elimination of impaired mitochondria, and facilitation of electrical conduction (Dick et al., 2019; Epelman et al., 2014; Hulsmans et al., 2017; Nicolás‐Ávila et al., 2020). Moreover, they inhibit monocyte and neutrophil recruitment, possibly by releasing IL‐10 and/or TGF‐β (Hilgendorf et al., 2014).

The regulatory molecules belonging to the CRM family have a significant impact on the coordination of repair and regeneration processes in the neonatal heart. They achieve this by controlling myocardial proliferation and angiogenesis, as evidenced by studies (Aurora et al., 2014; Lavine et al., 2014; Wang et al., 2019). In cases of chronic heart failure, CRMs respond to mechanical stretch through the TRPV4 pathway, triggering adaptations essential for sustaining cardiac output. These adaptations involve the reorganization of myocardial tissue and the development of coronary arteries (Revelo et al., 2021; Wong et al., 2021; Zaman et al., 2021). Despite the expression of high levels of MHC‐II and possession of antigen‐presenting capabilities by certain CRMs, the functional implications of this characteristic remain uncertain [8]. It is customary for CRMs to coexist with CCR2+ MHC‐IIhi macrophages, which originate from monocytes. CCR2+ MHC‐IIhi macrophages colonize the heart postnatally, approximately 2 weeks after birth. These macrophages exhibit potent inflammatory potential, secreting chemokines and facilitating the recruitment of neutrophils and monocytes through monocyte recruitment (Nahrendorf et al., 2007).

In addition, the heart harbors a limited number of Ly6C hiCCR2+ monocytes, which can be differentiated from cardiac macrophages based on their expression of MertK (Gautier et al., 2012). Moreover, immunosurveillance in the heart involves the participation of dendritic cells, mast cells, memory T cells, regulatory T cells, and intravascular B cells. Dendritic cells are believed to serve as guardians of self‐tolerance (Hart & Fabre, 1981; Satpathy et al., 2012). Nevertheless, the specific functions of these cellular populations within the heart remain incompletely understood.

To summarize, CRMs exhibit unique functionalities within the myocardium, engaging in diverse physiological processes, modulating inflammatory responses, and facilitating tissue healing and regeneration. Gaining a comprehensive understanding of the intricate interplay among distinct immune cell subsets within the cardiac milieu will enhance our comprehension of cardiac immunology and potentially pave the way for innovative therapeutic approaches targeting cardiovascular disorders.

## 3 CARDIAC AGING

The phenomenon of aging, characterized by the gradual decline in physiological function in the majority of living organisms, has historically captivated human curiosity and imagination. Presently, with the advancement of knowledge regarding the molecular and cellular mechanisms governing life and disease, aging has emerged as a prominent area of scientific inquiry.

**FIGURE 2:**
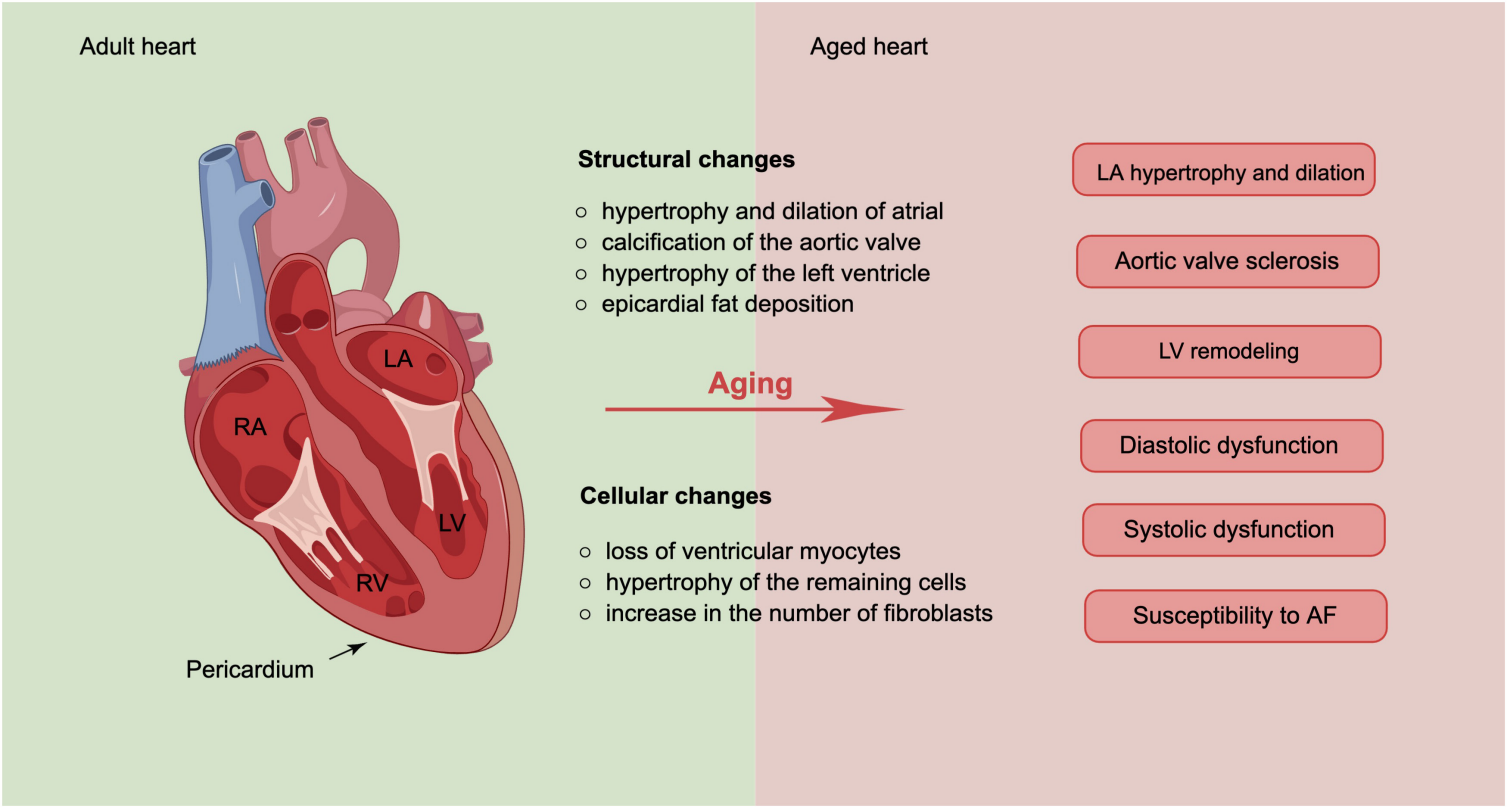
Overview of the age‐related changes to heart and aging‐related pathological processes.

In comparison to individuals aged 70–99, centenarians exhibit a reduced occurrence of cardiovascular diseases, including hypertension, myocardial infarction, angina, and diabetes (Galioto et al., 2008; Selim et al., 2005). This observation suggests that centenarians and their offspring possess a genetic or epigenetic makeup that confers protection against cardiovascular‐related mortality, thereby contributing to their longevity (Perls & Terry, 2003) (Figure 2).

Longitudinal studies, such as the Framingham Heart Study and the Baltimore Longitudinal Study on aging, have observed an age‐related increase in left ventricular wall thickness in apparently healthy adults. Additionally, the E/A ratio, a Doppler measurement indicating the ratio between early (E) and late (A) diastolic left ventricular filling, decreases with advancing age (Dai et al., 2009; Dai & Rabinovitch, 2009). This decline implies that a larger proportion of left ventricular filling takes place during late diastole rather than early diastole, which is clinically referred to as diastolic dysfunction or HFpEF. Age‐related left ventricular hypertrophy and diastolic dysfunction significantly increase (Dai et al., 2009). These changes may occur even in seemingly healthy individuals who do not have hypertension, indicating intrinsic cardiac aging.

Despite the relatively preserved systolic function, as indicated by ejection fraction, in the elderly population, there is a significant decline in exercise capacity and cardiovascular reserve with advancing age (Correia et al., 2002). The decrease in ejection fraction following maximal exercise and the decline in maximal heart rate contribute to the reduced exercise capacity observed in the elderly, which can be attributed to the effects of aging.

The augmented dependence on atrial contraction for left ventricular filling in diastolic dysfunction results in heightened atrial pressure, thereby exacerbating atrial hypertrophy, dilatation, and an escalated susceptibility to atrial fibrillation. This aligns with the notable rise in atrial fibrillation incidence among older individuals (Lakatta, 2003; Lakatta & Levy, 2003a, 2003b). In the geriatric cohort, atrial fibrillation detrimentally affects exercise capacity and predisposes individuals to HFpEF. HFpEF constitutes more than 50% of all heart failure instances in individuals aged 75 years and above, particularly without structural or ischemic heart disease.

Valvular changes associated with aging involve myxomatous degeneration, collagen deposition, and calcium deposition, resulting in valve sclerosis. Aortic valve sclerosis is present in 30%–80% of elderly individuals (Karavidas et al., 2010; Nassimiha et al., 2001; Stewart et al., 1997), detected by echocardiography as calcification of the annulus and leaflets of the aortic valve (Freeman & Otto, 2005; Otto et al., 1999). In the geriatric population, fibrosis and valvular calcification are prominent etiological factors in the pathogenesis of aortic stenosis, characterized by the progressive narrowing of the aortic valve orifice due to leaflet stiffening and calcification (Olsen et al., 2005). Consequently, this impedes efficient blood flow through the aortic valve, leading to the establishment of a pressure gradient between the aorta and the left ventricle. In order to sustain sufficient systolic function, the left ventricular walls undergo myocardial hypertrophy.

The cardiac aging‐related alterations in ventricular and valvular dynamics compromise the reserve capacity of the heart and reduce the threshold for functional impairment (Correia et al., 2002). As a result of these factors, the aging heart in geriatric individuals becomes more susceptible to stress and disease‐related challenges, resulting in a higher incidence of heart failure and cardiovascular mortality. Inflammaging, which is characterized by heightened basal inflammatory responses associated with aging (Franceschi & Campisi, 2014), leads to alterations in the immune composition of the cardiac tissue. Notably, there is a reduction in the replication of CRMs and a concomitant increase in the presence of proinflammatory CCR2+ MHC‐IIhi macrophages (Molawi et al., 2014). Furthermore, aging is accompanied by an elevation in the levels of circulating pro‐inflammatory cytokines and CCL2, indicating dysregulation of the innate immune system (Bruunsgaard et al., 2000; Chiao et al., 2011). The connection between inflammaging and the proliferation of mutations in hematopoietic stem cells, leading to the presence of mutated pro‐inflammatory myeloid cells in the cardiac immune system, has been emphasized in a recent study on clonal hematopoiesis (Pardali et al., 2020). In a mouse model of clonal hematopoiesis with Tet2 deficiency, there was an observed expansion of CCR2+ MHC‐IIhi macrophages in the cardiac region, but not CRMs, which exhibited an inflammatory reaction (Wang et al., 2020).

## 4 EMERGING ROLES OF MACROPHAGES IN CARDIAC DEVELOPMENT, CARDIAC AGING, AND OTHER RELATED CARDIOVASCULAR DISEASES MACROPHAGES IN CARDIAC DEVELOPMENT

During the process of heart development, the epicardium, which serves as the outer mesothelial layer, assumes a critical function in the recruitment of primitive yolk sac‐derived macrophages to the subepicardial space through signaling pathways (Stevens et al., 2016). This recruitment of embryonic macrophages aligns with notable morphological transformations in the developing heart, including chamber septation, valve remodeling, myocardial growth, electrical conduction system development, and the formation of coronary and lymphatic vasculature.

Tissue‐resident macrophages have been implicated in these processes through their involvement in engulfing dying cells, releasing cytokines, interacting with or recruiting progenitor cells, and undergoing trans‐differentiation into alternative cell types. Previous studies have demonstrated the functional significance of cardiac macrophages in valvular remodeling, normal conduction, and coronary development (Hulsmans et al., 2017; Leid et al., 2016; Shigeta et al., 2019). The development of the heart relies on the epicardium's secretion of paracrine signals, which stimulate the proliferation and maturation of cardiomyocytes, as well as support the expansion of the cardiac vasculature (Simões & Riley, 2018).

Tissue‐resident macrophages commonly coexist with coronary and lymphatic endothelium in the subepicardial space. Research has shown that macrophages play a crucial role in the growth and remodeling of cardiac lymphatic vessels. Macrophages colonize the embryonic heart prior to the initiation of lymphatic expansion and establish close associations and interactions with the adventitial surface and leading edges of lymphatic vessels. Their presence facilitates the growth and fusion of lymphatic vessels, ensuring sufficient coverage over the subepicardial surface (Cahill et al., 2021). Moreover, macrophages derived from the yolk sac are crucial for coronary maturation as they promote remodeling of the primitive coronary plexus, selectively expanding the perfused coronary vasculature (Leid et al., 2016).

Moreover, cardiac macrophages facilitate electrical conduction through the distal atrioventricular node, where conducting cells are interspersed with elongated macrophages expressing connexin 43. Through connexin 43‐containing gap junctions, these macrophages are coupled to spontaneously beating cardiomyocytes. Macrophages in the heart have a negative resting membrane potential and depolarize in synchrony with cardiomyocytes. According to computational modeling, macrophages increase the resting membrane potential of cardiomyocytes, thus accelerating their repolarization (Hulsmans et al., 2017).

In brief, the involvement of the epicardium and tissue‐resident macrophages is crucial in various aspects of heart development, including valvular remodeling, coronary and lymphatic vessel development, and electrical conduction. The epicardium functions by releasing paracrine signals that stimulate the proliferation of cardiomyocytes and aid in the expansion of the vasculature. On the other hand, macrophages contribute to the growth and remodeling of lymphatic vessels, promote the maturation of coronary vessels, and facilitate electrical conduction in specific regions of the heart.

## 5 ROLE OF MACROPHAGES IN CARDIAC AGING

Numerous age‐related alterations in cardiac function, encompassing myocardial sarcopenia, hypertrophy, vascular hyperpermeability, inflammation, fibrosis, and slight impairment of cardiac physiology, contribute to the development of cardiac morbidity and mortality (Lin et al., 2008; Lindsey et al., 2005; Yabluchanskiy et al., 2014). Prior investigations have demonstrated an age‐dependent increase in the population of cardiac macrophages in mice, exhibiting a positive correlation with age‐associated factors like MMPs and CCL2, which are implicated in cardiomyocyte hypertrophy and ventricular enlargement (Lindsey et al., 2005; Ma et al., 2015, 2012; Ma, Yuan, et al., 2018).

**FIGURE 3:**
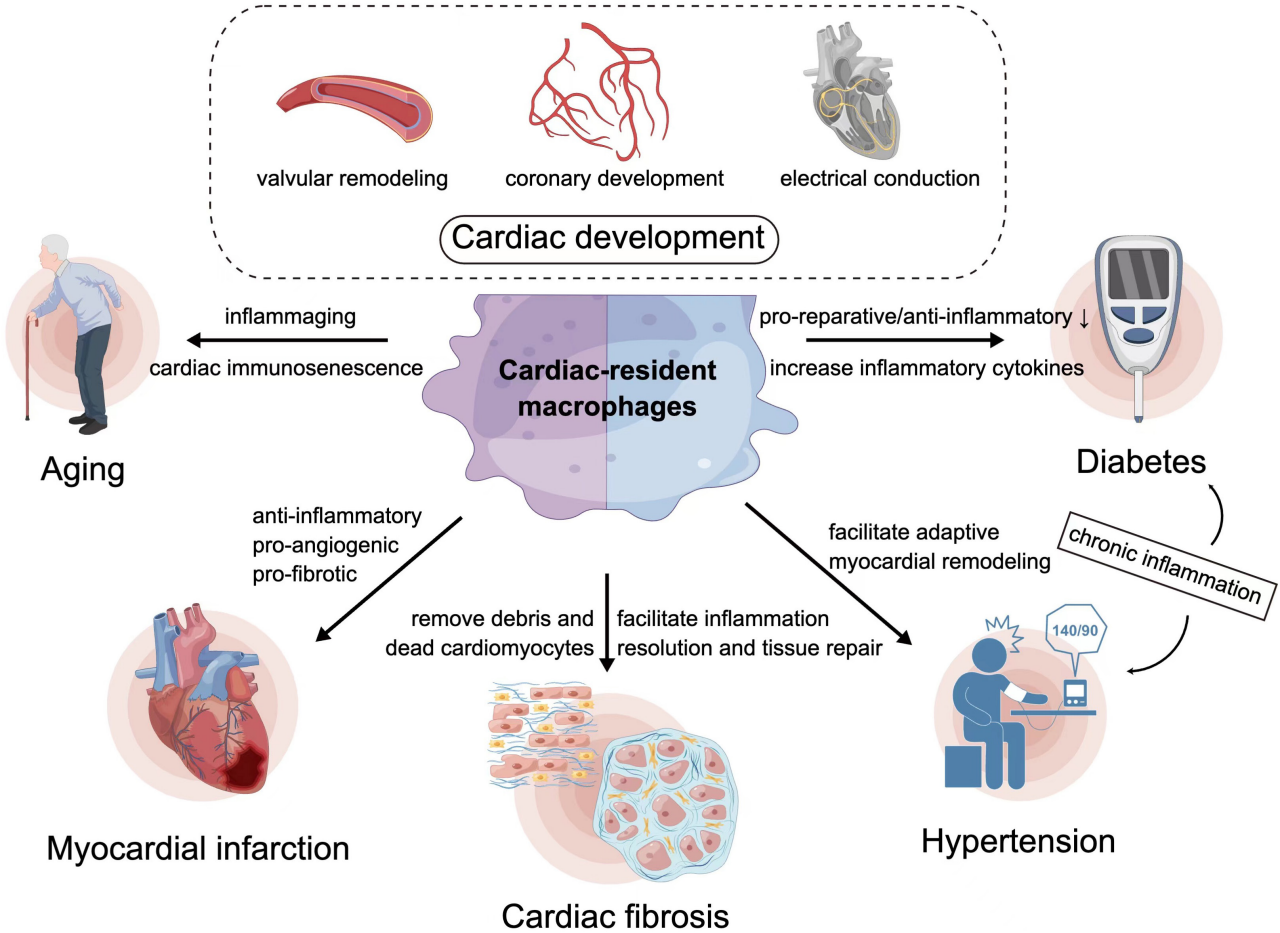
Macrophage involvement in age‐related changes to heart.

Based on flow cytometric analysis, it can be inferred that there is a noticeable increase in CD206+ cardiac resident macrophages and a corresponding decrease in CD206‐ CRMs as individuals age. This phenomenon appears to be influenced by MMP‐9 (Mouton et al., 2018; Toba et al., 2017). Furthermore, the process of aging has been associated with a rise in Ly6C+CCR2+ monocyte‐derived cardiac resident macrophages, commonly referred to as “inflammaging.” In addition, studies have shown that TLR2 signaling plays a role in ameliorating age‐related adverse cardiac remodeling and dysfunction (Meschiari et al., 2017; Spurthi et al., 2018; Wagner & Dimmeler, 2020). While there have been reports suggesting the classification of macrophages into activated and alternatively activated subsets, recent evidence indicates that further division of macrophage subsets is possible based on in vitro stimuli. For example, M1 macrophages can be categorized as M1a when stimulated with toll‐like receptors or as M1b subsets when stimulated with high‐mobility group protein B1 (Ben‐Mordechai et al., 2015). Subsets within macrophages exhibit distinct cell physiology. For instance, M1b demonstrates lower phagocytic activity compared to M1a. M2 macrophages can be further classified into M2a when stimulated with IL‐4 or IL‐13, M2b when stimulated with immune complexes in conjunction with IL‐1β, and M2c when stimulated with IL‐10, TGF‐β, or glucocorticoids (Gombozhapova et al., 2017; Martinez et al., 2008). Additionally, it is possible for different phenotypes to undergo reciprocal conversion under in vitro conditions. For instance, M1 macrophages can transition into the M2 phenotype upon stimulation with pro‐M2 factors, and vice versa (Pelegrin & Surprenant, 2009). Further investigation is required to elucidate the contribution of distinct subsets of CRMs and their mechanisms of renewal in cardiac aging, including the roles of self‐renewal and differentiation of blood monocytes (Figure 3).

Aging is characterized by the augmentation of the innate immune system's fundamental inflammatory response, commonly referred to as inflammaging, as well as the decline of the immune system in relation to cardiac function, known as cardiac immunosenescence. Inflammaging is a consequence of prolonged physiological stimulation of the innate immune system, which may incur damage during the aging process. Numerous factors contribute to the occurrence of inflammaging. The first contributing factor to inflammaging involves the accumulation of damaged macromolecules and cells (self‐debris) over time, resulting from heightened production and insufficient elimination. Additionally, chronic inflammation can arise from the presence of harmful substances originating from microbial constituents, such as those found in the oral or gut microbiota. Lastly, inflammaging may be attributed to cellular senescence, which is a cellular reaction to damage and stress (Franceschi & Campisi, 2014). The concept of immunosenescence was initially introduced by Roy Walford, encompassing the process of immune system deterioration and restructuring. This phenomenon leads to suboptimal vaccination results and heightened vulnerability to infections (Lian et al., 2020). Immunosenescence is marked by the incapacity to generate proficient humoral and cellular immune reactions against pathogens or vaccines, alongside a persistent low‐level inflammatory state known as “inflammaging.” This inflammatory state contributes to the dysregulation of various elements within the innate and adaptive immune systems (Franceschi et al., 2000; Xia et al., 2016).

Macrophages in the aging heart demonstrate compromised phagocytic capability and modified polarization, while innate immune cells exhibit reduced expression and functionality of TLRs (Khare et al., 1996; Mahbub et al., 2012; Spurthi et al., 2018; Wang & Shah, 2015). These processes, in conjunction with clonal hematopoiesis, contribute to the heightened susceptibility to cardiovascular diseases linked to the aging process. Nevertheless, the specific cellular states associated with aging and their correlation with acute or chronic inflammation remain incompletely comprehended, underscoring the significance of single‐cell omics studies as a valuable avenue for further investigation.

In summary, cardiac aging involves multiple changes in macrophage populations, inflammation, and immune function. Understanding these processes at the cellular level will provide valuable insights into the mechanisms underlying age‐related cardiovascular diseases.

## 6 ROLE OF MACROPHAGES IN MI

Following a myocardial infarction, the heart undergoes cardiac remodeling as a result of functional or structural stress on the cardiac system. This phenomenon holds significant importance in the progression of the disease. Initially, inflammatory cells, namely Ly6Chigh monocytes and M1 macrophages, are present (Figure 3). However, over time, these inflammatory cells are gradually substituted by anti‐inflammatory cells, specifically Ly6Clow monocytes and M2 macrophages. Following the resolution of the inflammatory phase, the macrophages assume responsibility for wound healing by producing various factors, including IL‐10 (anti‐inflammatory), VEGF, PDGF, and IGF‐1 (pro‐angiogenic), as well as TGF‐β1 and fibronectin (pro‐fibrotic). These factors aid in the reconstruction of the vascular supply and the repair of necrotic tissue (Atri et al., 2018; Lafuse et al., 2020). While this remodeling initially enhances cardiac performance, it can have detrimental consequences, such as cardiomyocyte death, compromised ventricular wall integrity, impaired ventricular function, and cardiac fibrosis, ultimately contributing to the development of heart failure. Despite the abundance of macrophages in infarcted hearts and their significant role in inflammation, the precise mechanism by which these receptors interact to suppress inflammatory signals and resolve leukocyte infiltration remains unclear. In the context of post‐myocardial infarction inflamed heart tissue, distinct functional subpopulations of macrophages, such as regulatory and reparative macrophages, have been identified. However, the temporal dynamics of macrophage populations following infarction are not well understood (Khare et al., 1996). It is uncertain whether new subpopulations emerge to replace the original macrophages or if the original cells undergo transformation into different phenotypes. Recent research has focused on the utilization of macrophages as a therapeutic approach for addressing the progressive adverse remodeling of the heart following myocardial infarction. Specifically, the enhancement of cardiac function and mitigation of detrimental ventricular remodeling have been observed through the promotion of survival in Ly6Clow macrophages and their CCR2‐MHCII low subsets via BDNF‐mediated pathways, facilitated by environmental eustress (Mahbub et al., 2012). A separate study has provided evidence that the absence of EDIL3 enhances the healing process of the heart by decreasing the polarization of M1 macrophages. Furthermore, the removal of TAZ encourages the inhibition of IL‐6 and the expression of Arg1, resulting in a decrease in ventricular hypertrophy and fibrosis, as well as an increase in angiogenesis (Renshaw et al., 2002). In addition, it has been discovered that macrophages contribute to the growth of lymphatic vessels in the myocardium and aid in the reduction of cardiac damage following a myocardial infarction by releasing VEGFC (Atri et al., 2018; D'Amore & Alcaide, 2022). Understanding the regulation of macrophage polarization and their effects on cardiac function post‐MI can provide new insights into potential therapeutic strategies. While transitioning from a quiescent to an activated state, macrophages undergo a global rewiring of metabolic pathways (Lafuse et al., 2020). To meet the bioenergetic and biosynthetic demands of the inflammation process, pro‐inflammatory macrophages switch from oxidative metabolism to glycolysis (Mosser & Edwards, 2008). Activated macrophages rapidly generate adenosine triphosphate (ATP), leading to the production of NADPH for bactericidal activity through the pentose phosphate pathway (Bai et al., 2022). In pro‐inflammatory macrophages, mitochondrial oxidative metabolism is suppressed, and there is a disruption and rewiring of tricarboxylic acid cycle flux (Mia et al., 2020; Wei et al., 2022). In the context of myocardial infarction, infiltrating monocytes/macrophages mount a similar inflammatory response. This results in activated macrophages exhibiting glycolytic polarization due to the release of damage‐associated molecular patterns by dead cells and subsequent activation of toll‐like receptors (Liu et al., 2019). Macrophages MI exhibit a significant reprogramming of mitochondrial genes, which is indicative of their active involvement in tissue repair during the transition from injury to the infarct zone. This finding underscores the pivotal role played by mitochondrial function in shaping macrophage phenotype and facilitating cardiac remodeling following MI.

The clearance of apoptotic cells by immune cells plays a crucial role in resolving inflammation and promoting tissue repair after myocardial infarction (Gordon, 2016; Serhan & Savill, 2005; Wan et al., 2013). Phagocytic macrophages involved in the process of efferocytosis undergo a metabolic shift characterized by heightened oxygen consumption, upregulated FAO and OXPHOS, and reduced glycolysis (Park et al., 2011). These metabolic alterations coincide with the decrease in cytokines and damage‐associated molecular patterns (DAMPs) in the microenvironment as inflammation subsides. The involvement of mitochondrial function in cardiac repair and remodeling has been evidenced by the impaired wound healing and heightened cardiac rupture observed in mice with myeloid‐specific deficiency of Complex III in the electron transport chain(B. Zhang et al., 2018). Nevertheless, additional investigation is required to comprehend the underlying mechanisms and therapeutic implications of mitochondrial metabolism in macrophage phenotype and cardiac remodeling.

In the context of myocardial infarction, the process of cardiac remodeling encompasses intricate interplays among macrophages, inflammation, and metabolic pathways. By focusing on macrophages and manipulating their polarization and metabolism, there exists the possibility of implementing strategies aimed at ameliorating adverse ventricular remodeling and enhancing cardiac function subsequent to MI.

## 7 CROSSTALK BETWEEN MACROPHAGES AND CARDIOMYOCYTES

**FIGURE 4:**
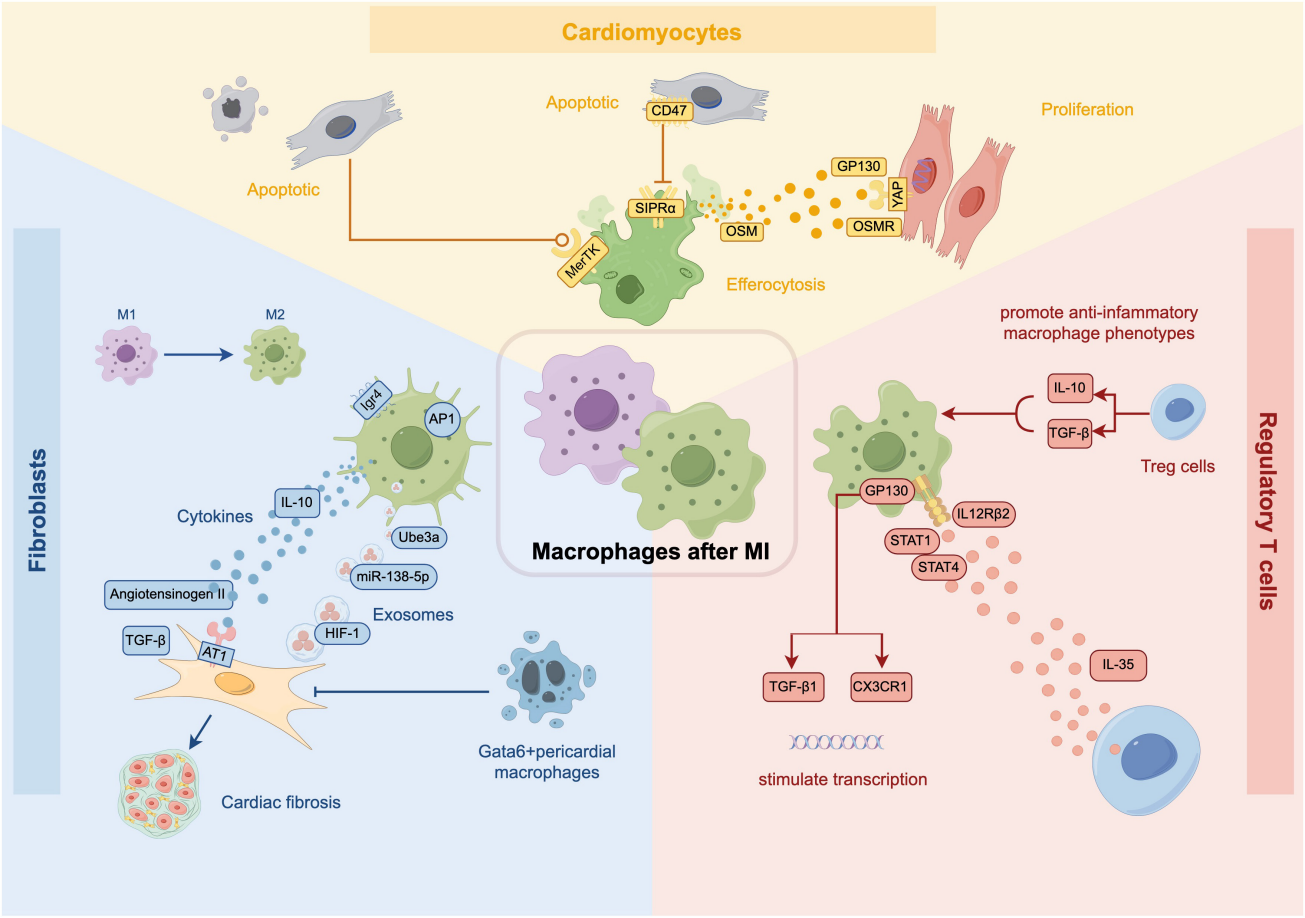
Crosstalk between macrophages and other types of cells, including cardiomyocytes, fibroblasts, and Treg cells.

Following myocardial infarction (MI), the process of efferocytosis performed by macrophages assumes significance in the effective removal of deceased and apoptotic cardiac myocytes (CMs). Numerous investigations have successfully identified distinct efferocytosis receptors present on macrophages. Notably, several studies have demonstrated the capacity of macrophages to identify CMs through the utilization of the MerTK receptor (Cai et al., 2017; Zagórska et al., 2014). The impairment of cardiac metabolic function occurs when either macrophages or MerTK are ablated (Nicolás‐Ávila et al., 2020). Furthermore, the proteins CD47 and CD72, which are associated with integrin, exert an influence on the intercommunication between macrophages and cardiac myocytes after myocardial infarction (MI). The upregulation of CD47 expression hampers the process of efferocytosis through the CD47‐SIRPα axis (Nicolás‐Ávila et al., 2020). Additionally, it has been observed that macrophages play a significant role in facilitating cardiac muscle (CM) proliferation and regeneration following myocardial infarction. Notably, the hearts of neonatal mice have demonstrated the ability to fully regenerate post‐MI through the activation of several downstream signaling pathways, including Hippo‐Yap, Jak1‐STAT3, and Notch (Bassat et al., 2017; Han et al., 2015; Xin et al., 2013). Yan et al. discovered that macrophages possess the ability to release oncostatin M, thereby exerting regulatory control over cardiac myocyte proliferation and cardiac regeneration via the activation of the gp130/Src/Yap‐Notch and Yap‐ctgf/Areg signaling pathways (Li et al., 2020) (Figure 4).

## 8 CROSSTALK BETWEEN MACROPHAGES AND FIBROBLASTS

During the wound‐healing phase subsequent to myocardial infarction, macrophages that are recruited have the capability to generate angiotensinogen II, thereby activating the classical renin‐angiotensin‐aldosterone system and inducing an upregulation of TGF‐β1. This regulatory process effectively governs the deposition of matrix proteins and inhibits matrix degeneration, ultimately facilitating tissue repair (Weber et al., 2013). A recent study has provided evidence that Igr4, a G protein‐coupled receptor containing leucine‐rich repeats, has the ability to enhance AP‐1 activation within inflammatory macrophages through pathways mediated by CREB (Huang et al., 2020). In recent years, a multitude of scholarly investigations have been conducted to examine the significance of macrophage‐derived exosomes in the context of fibrosis. It has been observed that M2‐like macrophages possess the ability to stimulate the circUbe3a/miR‐138‐5p/Rhoc signaling axis, thereby facilitating the progression of cardiac fibrosis subsequent to myocardial infarction (Y. Wang et al., 2021). Abe et al. discovered that following myocardial infarction, Ly‐6Chigh macrophages release hypoxia‐inducible factor 1 (HIF‐1) in order to modulate the OSM gene, consequently impeding the activation of cardiac fibroblasts through the ERK1/2‐SMAD2‐TGFβ1 axis (Abe et al., 2019). Furthermore, the involvement of Gata6 + pericardial macrophages (GPCMs) in the progression of cardiac fibrosis subsequent to myocardial infarction (MI) was observed. Deniset et al. discovered a gradual recruitment of GPCMs to the infarcted site following MI, accompanied by alterations in their phenotypic characteristics. Notably, Gata6‐knockout mice exhibited an augmented presence of detrimental fibrosis within the infarcted region (Deniset et al., 2019) (Figure 4).

## 9 CROSSTALK BETWEEN MACROPHAGES AND T LYMPHOCYTES

T lymphocyte populations can be broadly categorized into two subsets: helper CD4+ T lymphocytes and cytotoxic CD8+ T lymphocytes. The former subset plays a role in myocardial infarction through communication with macrophages. CD4+ T cells after MI can also be classified into two subsets based on Foxp3 expression: “effector” (Tef; Foxp3‐) and “regulatory” (Treg; Foxp3+). Treg cells (secreting IL‐10 and TGF‐β) promote anti‐inflammatory macrophage phenotypes, thus influencing the process of healing and scar formation (Ramos et al., 2016). Jia et al. observed that following myocardial infarction, regulatory T cells (Treg cells) exhibit an increased secretion of interleukin‐35 compared to normal physiological conditions. This heightened IL‐35 production stimulates the transcription of CX3CR1 (C‐X3‐C motif chemokine receptor 1) and transforming growth factor‐beta 1 in macrophages. This activation is mediated through the GP130 and IL12Rβ2 signaling pathways, as well as the phosphorylation of STAT1 and STAT4. Consequently, the enhanced expression of CX3CR1 and TGF‐β1 promotes the survival of Ly‐6Clow macrophages and facilitates the deposition of extracellular matrix (Jia et al., 2019) (Figure 4).

## 10 ROLE OF MACROPHAGES IN IN CARDIAC FIBROSIS

The accumulation of extracellular matrix (ECM) proteins within the cardiac interstitium is a defining characteristic of cardiac fibrosis, a condition that arises from persistent injury to the myocardium. This pathological process gives rise to myocardial wall thickening, as well as systolic and diastolic dysfunction, ultimately impairing overall cardiac performance. Under normal circumstances, the ECM serves to provide structural support to cardiac cells, ensuring the integrity and functionality of the heart (Fan et al., 2012). Additionally, the ECM facilitates the transmission of electrical conduction and contractile force throughout the cardiomyocytes within the entire organ. Furthermore, the ECM serves as a reservoir for latent growth factors (Kong et al., 2014). Myocardial injury can be induced by either sterile or non‐sterile inflammation resulting from an infection. The resolution of inflammation necessitates an anti‐inflammatory response, subsequently activating profibrotic signaling (Frangogiannis, 2008). Furthermore, interstitial, and perivascular fibrosis can arise from pathophysiological stimuli, including pressure overload, volume overload, metabolic dysfunction, and aging, even in the absence of myocardial inflammation (Figure 3).

Both resident embryonic macrophages and recruited macrophages are responsible for removing debris and dead cardiomyocytes during the initial inflammatory phase, while matrix metalloproteinases break down the extracellular matrix. Once enough clearance is accomplished, a proliferative phase is initiated, resulting in the transformation of fibroblasts into myofibroblasts. Myofibroblasts have a significant role in producing collagen and other matrix proteins to compensate for cellular loss in different heart failure‐related conditions (Kong et al., 2014; Murphy et al., 2015). During the proliferative phase, both immune and nonimmune cells in the injured myocardium release mediators that have anti‐inflammatory and pro‐fibrotic properties. These mediators include lactoferrin, annexin A1, TGF‐β1, IL‐10, lipoxins, resolvins, and MMPs. They play a crucial role in resolving inflammation and initiating remodeling processes (DeLeon‐Pennell et al., 2017; Frangogiannis, 2012; Huynh et al., 2002; Soehnlein & Lindbom, 2010). TGF‐β1, which is typically bound to the ECM, becomes activated and facilitates myofibroblast trans‐differentiation through signaling pathways involving Smad3 and the expression of smooth muscle actin (DeLeon‐Pennell et al., 2017). To ensure the preservation of structural integrity and mitigate the occurrence of wall rupture and myocardial dysfunction, activated myofibroblasts exhibit heightened secretion of collagens and other extracellular matrix proteins. Nevertheless, an excessive deposition of ECM results in ventricular stiffness, impaired contractile function, and an augmented susceptibility to arrhythmogenesis and mortality. Additionally, inflammation and fibrosis in perivascular regions contribute significantly to pathological remodeling. During the maturation stage, a scar is formed with a cross‐linked extracellular matrix subsequent to the proliferative stage. Apoptosis occurs in myofibroblasts, temporary disintegration of microvasculature takes place, and tissue‐resident macrophages play a role in facilitating inflammation resolution and tissue repair. The suppression of monocyte recruitment to the injured adult heart is observed due to reduced inflammation and accelerated repair, while the embryonic macrophage population remains unaffected. In conclusion, the intricate interaction among inflammatory cells, myofibroblasts, extracellular matrix remodeling, and macrophages plays a pivotal role in the advancement and resolution of cardiac fibrosis.

## 11 ROLE OF MACROPHAGES IN CARDIAC REMODELING IN HYPERTENSION AND DIABETIC CARDIOMYOPATHY

Diabetes and hypertension, frequently coexisting, are significant risk factors for cardiovascular diseases, encompassing myocardial infarction and heart failure. These conditions are distinguished by persistent, mild inflammation, which contributes to adverse alterations in the structure and function of the heart. Inflammation assumes a pivotal role in the heart's reaction to injury and adaptive remodeling (DeBerge et al., 2019) (Figure 3). Nonetheless, inflammation can impede the adaptive response and lead to cardiac impairment. The activation of macrophages by diabetes and hypertension can lead to chronic inflammation, prompting them to adopt an inflammatory phenotype. While macrophages play a vital role in cardiac remodeling, an imbalance between pro‐inflammatory and anti‐inflammatory phenotypes can lead to excessive inflammation and subsequent cardiac damage (Mouton et al., 2020). Under normal circumstances, a healthy heart primarily utilizes fatty acids as its main energy source through mitochondrial oxidative phosphorylation. Conversely, in cases of decompensated heart failure, the heart predominantly relies on glycolysis (Berthiaume et al., 2012; Dodd et al., 2012). In the context of heart failure, the activation of the HIF‐1α transcription factor in this particular state results in the transcription of genes that are linked to glycolysis and inflammation, thereby contributing to adverse remodeling (Boutens et al., 2018; Lauterbach & Wunderlich, 2017). Additionally, HIF‐1α can be induced by nonhypoxic mechanisms associated with obesity and hypertension, including inflammatory cytokines, hyperglycemia, activation of toll‐like receptor 4 by saturated fatty acids, and oxidized low‐density lipoprotein (Groh et al., 2018; Lancaster et al., 2018; Tannahill et al., 2013; Wu et al., 2014). Furthermore, individuals with diabetes exhibit increased vulnerability to bacterial infections and impaired wound healing. The impaired pro‐reparative/anti‐inflammatory functions of macrophages and the heightened expression of long‐chain acyl‐CoA synthetase (Kanter et al., 2012; Pavlou et al., 2018; Wicks et al., 2014), may account for this phenomenon. Additionally, macrophages are susceptible to protein glycation and the formation of advanced glycation end products (AGEs) in response to elevated glucose levels. This activation of the NF‐κB pathway subsequently induces the production of inflammatory cytokines (Jin et al., 2015; Mishra et al., 2017). Furthermore, it has been observed that macrophages derived from individuals with coronary artery disease, which is frequently associated with obesity and hypertension, display notably increased levels of IL‐1β and TNF‐α compared to those with inflammatory vascular diseases (Watanabe et al., 2018). Consequently, in the presence of hyperglycemia, macrophages seem to enhance their uptake and utilization of glucose, resulting in heightened production of inflammatory cytokines and the induction of an inflammatory phenotype (Watanabe et al., 2018). Moreover, the presence of free fatty acids, lipid mediators, and adipokines can also contribute to the promotion of this inflammatory phenotype (Francisco et al., 2018; Kain et al., 2018; Namgaladze & Brüne, 2016).

Cardiac macrophages have a significant impact on the regulation of cardiac remodeling in cases of obesity and hypertension. Normally, the healthy heart harbors a limited number of M2‐like macrophages, derived from embryonic cells, which are deemed to have a protective function (Bajpai et al., 2019; Ben‐Mordechai et al., 2013; Ma, Mouton, & Lindsey, 2018). Nevertheless, in the absence of injury, CCR2+ monocyte‐derived macrophages progressively replace these M2‐like macrophages as individuals age (Bajpai et al., 2019). In a mouse model of pressure overload, resident M2‐like macrophages demonstrate their ability to facilitate adaptive myocardial remodeling in response to mechanical stress. However, the infiltration of CCR2+ monocytes has been found to contribute to maladaptive remodeling during the transition to decompensated heart failure (Liao et al., 2018). EAT is known to contain various immune cells such as macrophages, neutrophils, and lymphocytes, which may play a role in protecting against heart infections (Horckmans et al., 2018). Notably, during exercise, myokines released by the heart muscles have been shown to influence the phenotype of EAT macrophages, promoting a protective M2‐like phenotype (Aldiss et al., 2017). Conversely, in the context of cardiac injury, EAT can serve as a significant source of inflammatory macrophages due to hypoxia‐induced activation, leading to their easy infiltration into the myocardium (Guzzardi & Iozzo, 2019; Vianello et al., 2019). In mice, the role of EAT in inflammation following myocardial infarction has been found to be significant (Vianello et al., 2019). Moreover, in patients with MI, an augmented thickness of EAT has been linked to visceral obesity and cardiac fibrosis (Gruzdeva et al., 2018). Furthermore, in cases of severe cardiac injury, necrotic myocytes have the ability to recruit and activate macrophages through the release of damage‐associated molecular patterns (Ma, Mouton, & Lindsey, 2018; Mouton et al., 2018). Additionally, myocytes with impaired mitochondria can release damage‐associated molecular patterns, which subsequently enhance the secretion of pro‐inflammatory chemokines and cytokines (Zhang et al., 2010). The occurrence of endothelial cell injury, induced by pressure‐induced shear stress and mechanical stretch, is a significant factor in the development of cardiac inflammation in individuals with hypertension. This injury prompts an augmented production of ROS and hampers NO signaling, thereby potentially attracting neighboring macrophages (Savoia et al., 2011).

Extensive research has been conducted on the involvement of macrophages in cardiac remodeling subsequent to myocardial infarction, as evidenced by numerous studies (Andreadou et al., 2019; Ben‐Mordechai et al., 2013; Chen & Frangogiannis, 2018; Dick et al., 2019). However, the exploration of macrophage metabolism in this context remains constrained. Notably, a mouse model of myocardial infarction demonstrates a significant upregulation of glycolytic genes, including GAPDH, in macrophages within the infarcted region on the first day. Conversely, mitochondrial genes, such as succinate dehydrogenase, exhibit an increase on the third day following myocardial infarction (Mouton et al., 2018), suggesting that the cardiac microenvironment is hypoxic on the first day but becomes reoxygenated by Day 3 due to vasculogenesis (Mouton et al., 2018, 2019). In a rat model of myocardial infarction, the utilization of a glycolysis inhibitor resulted in a reduction of glycolysis and inflammation in cardiac macrophages, ultimately leading to an improvement in left ventricular function (Lewis et al., 2018). The underlying mechanism involves the process of efferocytosis, which enhances the intracellular supply of fatty acids, thereby promoting mitochondrial fatty acid oxidation and inducing a shift in macrophage polarization toward M2 phenotypes (Zhang et al., 2019). This M2‐like polarization has been found to contribute to cardiac fibrosis in mice with hypertension and diastolic dysfunction, but its initiation is dependent on the initial infiltration and expansion of inflammatory macrophages (Hulsmans et al., 2018). Furthermore, it has been previously discussed that resident macrophages in the mouse heart, which do not express CCR2, can be categorized into two groups based on their MHCII expression (Epelman et al., 2014). Additionally, CCR2+ cardiac monocyte‐derived macrophages can originate from infiltrating Ly6Chi (M1‐like) or Ly6Clow (M2‐like) monocytes (Liao et al., 2018; Nahrendorf et al., 2007). This suggests that there might be variations in metabolic profiles not only between the M1 and M2 paradigms, but also between resident macrophages and infiltrating monocytes. However, the field of immunometabolism is still in its nascent stage and predominantly relies on the classification of M1/M2 phenotypes, with future investigations focusing on exploring different subsets of cardiac macrophages in the context of cardiac remodeling.

## 12 MACROPHAGE‐TARGETED THERAPIES

Extensive research has been conducted on the subject of macrophage‐targeted therapies in heart failure; nevertheless, the development of effective clinical treatments based on this knowledge remains elusive. Macrophages assume a crucial role in the mediation of tissue damage and the formation of fibrotic scars in heart failure. Leveraging macrophages as therapeutic targets holds the potential to mitigate the deleterious impacts of the innate immune system, while concurrently preserving its indispensable functions. The implementation of macrophage‐targeted therapy necessitates the design of nanoparticles and nano‐based drug delivery systems due to the phagocytic capabilities exhibited by macrophages. The regulation of inflammatory monocyte migration, which gives rise to classically activated macrophages and contributes to inflammatory diseases, is governed by the CCR2 marker. Leuschner et al. (2011) devised siRNA nano‐molecules with a specific affinity for monocytes to effectively suppress CCR2 mRNA expression in inflammatory monocytes, thereby selectively impeding their migration. These nano‐molecules were successfully internalized by monocytes and exhibited accumulation in the spleen and bone marrow of mice upon systemic administration. Notably, the degradation of CCR2 mRNA in monocytes resulted in diminished accumulation at inflammatory sites. This therapeutic approach demonstrated a reduction in infarct size following coronary artery occlusion, a decrease in atherosclerotic plaques, and a decline in tumor‐associated macrophages in mouse models.

Viral infection and autoimmune myocarditis are substantial contributors to heart failure in adolescents, thus necessitating the implementation of macrophage‐targeted therapy. It has been documented that individuals with myocarditis exhibit heightened levels of CSF‐1 expression. CSF‐1, which is synthesized by cells of the mononuclear phagocyte system, exerts influence over the genesis and progression of monocytes/macrophages via the CSF‐1R. Meyer et al. (2018) employed nanoparticle‐encapsulated siRNA to suppress the CSF‐1 axis, resulting in the alleviation of acute inflammatory heart injury and the mitigation of long‐term repercussions associated with acute myocardial damage. This therapeutic strategy demonstrated efficacy in the treatment of viral and autoimmune myocarditis.

Due to their participation in inflammatory heart tissue and phenotypic plasticity, macrophages have been recognized as a distinct target for the treatment of cardiovascular diseases. Potential therapies targeting macrophages in heart failure may utilize macrophage markers to deliver treatment specific to the affected tissues. Furthermore, recent advancements in comprehending the epigenetic programming of fibrosis and HF, including macrophage polarization, present encouraging prospects for intervening in macrophage epigenetics through genetic or pharmacological means (Davis & Gallagher, 2019; Liu & Tang, 2019). The CRISPR technology, which enables enduring genetic modifications in cells, could potentially be utilized to modify macrophage polarization and functional phenotype (Stoppe et al., 2018).

The potential protective function of macrophage migration inhibitory factor has been documented following cardiac surgery. Despite the limited research on macrophages and cardiac surgery at present, this field exhibits promising prospects for future exploration.

Despite the considerable progress made in modern medicine, heart failure continues to have a substantial impact on morbidity and mortality rates. The management of HF is contingent upon identifying its root cause, as failure to address it promptly can lead to rapid disease progression and a decline in the patient's quality of life (McDonagh et al., 2022). Stem cell therapy, an emerging technology, has demonstrated potential as a therapeutic strategy for both prevention and treatment of cardiovascular diseases (Goradel et al., 2018). In this context, macrophages play a crucial role in the implementation of cardiac stem cell therapy. Numerous studies have provided evidence that, subsequent to ischemia–reperfusion injury, the cardiac function in mice undergoes enhancement via an acute sterile immune response, which entails the activation of CCR2+ and CX3CR1+ macrophages, rather than the generation of new cardiomyocyte (Vagnozzi et al., 2020). This specific macrophage response leads to improved activity of cardiac fibroblasts, decreased extracellular matrix content, and augmented mechanical properties within the ischemic region, thereby presenting a novel mechanism for cell‐mediated cardiac protection. To comprehensively comprehend the therapeutic implications of these findings, further research is imperative. The reciprocal regulation between macrophages and stem cells is observed in the context of cardiac regeneration in the injured heart. Previous research has demonstrated that the interaction between BMMSCs and immune cells, specifically macrophages, leads to a shift in macrophage polarization toward an anti‐inflammatory state (Cho et al., 2014; Hare et al., 2012). This effect is further enhanced when macrophages are primed with BMMSCs, as evidenced by Lim et al. (2018) in their study utilizing a rat model of myocardial infarction. The administration of a coculture of bone marrow‐derived mesenchymal stem cells and bone marrow‐derived macrophages (BMDMs) via injection led to a notable augmentation in the prevalence of M2 macrophages, thereby yielding enhanced cardiac functionality, improved angiogenesis, and diminished cardiac fibrosis. These findings suggest that macrophages possess considerable therapeutic capabilities, and BMDMs that have been primed with BMMSCs serve as an efficacious adjunctive therapy for cardiac restoration.

## 13 CONCLUSION

With the growing proportion of elderly individuals in the global population, it becomes imperative to comprehend the alterations in cardiac structure and function that occur as part of the natural aging process. Cardiac tissue‐resident macrophages represent the predominant immune cell population within the heart. Therefore, gaining insight into the mechanistic underpinnings of CRM involvement during the aging process holds significant importance for the development of novel therapeutic interventions aimed at enhancing patient outcomes.

## AUTHOR CONTRIBUTIONS

Jiayu Li and Yanguo Xin wrote the main manuscript; Jingye Li and Zhaojia Wang prepared the figures; Weiping Li and Hongwei Li reviewed the manuscript. Hongwei Li offered the idea of the manuscript.

## FUNDING INFORMATION

This work was supported by the National Key R&D Program of China (Grant No. 2021ZD0111000); the National Natural Science Foundation of China (Grant No. 82070357) and Beijing Key Clinical Subject Program.

## CONFLICT OF INTEREST STATEMENT

All authors declare that there is no conflict of interest.

## CONSENT FOR PUBLICATION

All authors offer consent for publication.

## Data Availability

All related materials are available from Hongwei Li.

## References

[acel14008-bib-0001] Abe, H. , Takeda, N. , Isagawa, T. , Semba, H. , Nishimura, S. , Morioka, M. S. , Nakagama, Y. , Sato, T. , Soma, K. , Koyama, K. , Wake, M. , Katoh, M. , Asagiri, M. , Neugent, M. L. , Kim, J. W. , Stockmann, C. , Yonezawa, T. , Inuzuka, R. , Hirota, Y. , … Komuro, I. (2019). Macrophage hypoxia signaling regulates cardiac fibrosis via Oncostatin M. Nature Communications, 10(1), 2824. 10.1038/s41467-019-10859-w PMC6597788 31249305

[acel14008-bib-0002] Aldiss, P. , Davies, G. , Woods, R. , Budge, H. , Sacks, H. S. , & Symonds, M. E. (2017). 'Browning' the cardiac and peri‐vascular adipose tissues to modulate cardiovascular risk. International Journal of Cardiology, 228, 265–274. 10.1016/j.ijcard.2016.11.074 27865196 PMC5236060

[acel14008-bib-0003] Andreadou, I. , Cabrera‐Fuentes, H. A. , Devaux, Y. , Frangogiannis, N. G. , Frantz, S. , Guzik, T. , Liehn, E. A. , Gomes, C.P. , Schulz, R. , & Hausenloy, D. J. (2019). Immune cells as targets for cardioprotection: New players and novel therapeutic opportunities. Cardiovascular Research, 115(7), 1117–1130. 10.1093/cvr/cvz050 30825305 PMC6529904

[acel14008-bib-0004] Atri, C. , Guerfali, F. Z. , & Laouini, D. (2018). Role of human macrophage polarization in inflammation during infectious diseases. International Journal of Molecular Sciences, 19(6), 1801. 10.3390/ijms19061801 PMC6032107 29921749

[acel14008-bib-0005] Aurora, A. B. , Porrello, E. R. , Tan, W. , Mahmoud, A. I. , Hill, J. A. , Bassel‐Duby, R. , Sadek, H. A. , & Olson, E. N. (2014). Macrophages are required for neonatal heart regeneration. The Journal of Clinical Investigation, 124(3), 1382–1392. 10.1172/jci72181 24569380 PMC3938260

[acel14008-bib-0006] Bai, P. Y. , Chen, S. Q. , Jia, D. L. , Pan, L. H. , Liu, C. B. , Liu, J. , Luo, W. , Yang, Y. , Sun, M. Y. , Wan, N. F. , Rong, W. W. , Sun, A. J. , & Ge, J. B. (2022). Environmental eustress improves postinfarction cardiac repair via enhancing cardiac macrophage survival. Science Advances, 8(17), eabm3436. 10.1126/sciadv.abm3436 35476440 PMC9045726

[acel14008-bib-0007] Bajpai, G. , Bredemeyer, A. , Li, W. , Zaitsev, K. , Koenig, A. L. , Lokshina, I. , Mohan, J. , Ivey, B. , Hsiao, H. M. , Weinheimer, C. , Kovacs, A. , Epelman, S. , Artyomov, M. , Kreisel, D. , & Lavine, K. J. (2019). Tissue resident CCR2‐ and CCR2+ cardiac macrophages differentially orchestrate monocyte recruitment and fate specification following myocardial injury. Circulation Research, 124(2), 263–278. 10.1161/circresaha.118.314028 30582448 PMC6626616

[acel14008-bib-0009] Bassat, E. , Mutlak, Y. E. , Genzelinakh, A. , Shadrin, I. Y. , Baruch Umansky, K. , Yifa, O. , Kain, D. , Rajchman, D. , Leach, J. , Riabov Bassat, D. , Udi, Y. , Sarig, R. , Sagi, I. , Martin, J. F. , Bursac, N. , Cohen, S. , & Tzahor, E. (2017). The extracellular matrix protein agrin promotes heart regeneration in mice. Nature, 547(7662), 179–184. 10.1038/nature22978 28581497 PMC5769930

[acel14008-bib-0010] Benjamin, E. J. , Blaha, M. J. , Chiuve, S. E. , Cushman, M. , Das, S. R. , Deo, R. , de Ferranti, S. D. , Floyd, J. , Fornage, M. , Gillespie, C. , Isasi, C. R. , Jiménez, M. C. , Jordan, L. C. , Judd, S. E. , Lackland, D. , Lichtman, J. H. , Lisabeth, L. , Liu, S. , Longenecker, C. T. , … Muntner, P. (2017). Heart disease and stroke Statistics‐2017 update: A report from the American Heart Association. Circulation, 135(10), e146–e603. 10.1161/cir.0000000000000485 28122885 PMC5408160

[acel14008-bib-0011] Ben‐Mordechai, T. , Holbova, R. , Landa‐Rouben, N. , Harel‐Adar, T. , Feinberg, M. S. , Abd Elrahman, I. , Blum, G. , Epstein, F. H. , Silman, Z. , Cohen, S. , & Leor, J. (2013). Macrophage subpopulations are essential for infarct repair with and without stem cell therapy. Journal of the American College of Cardiology, 62(20), 1890–1901. 10.1016/j.jacc.2013.07.057 23973704

[acel14008-bib-0012] Ben‐Mordechai, T. , Palevski, D. , Glucksam‐Galnoy, Y. , Elron‐Gross, I. , Margalit, R. , & Leor, J. (2015). Targeting macrophage subsets for infarct repair. Journal of Cardiovascular Pharmacology and Therapeutics, 20(1), 36–51. 10.1177/1074248414534916 24938456

[acel14008-bib-0013] Berthiaume, J. M. , Young, M. E. , Chen, X. , McElfresh, T. A. , Yu, X. , & Chandler, M. P. (2012). Normalizing the metabolic phenotype after myocardial infarction: Impact of subchronic high fat feeding. Journal of Molecular and Cellular Cardiology, 53(1), 125–133. 10.1016/j.yjmcc.2012.04.005 22542451 PMC3372615

[acel14008-bib-0014] Boutens, L. , Hooiveld, G. J. , Dhingra, S. , Cramer, R. A. , Netea, M. G. , & Stienstra, R. (2018). Unique metabolic activation of adipose tissue macrophages in obesity promotes inflammatory responses. Diabetologia, 61(4), 942–953. 10.1007/s00125-017-4526-6 29333574 PMC6448980

[acel14008-bib-0015] Bruunsgaard, H. , Skinhøj, P. , Pedersen, A. N. , Schroll, M. , & Pedersen, B. K. (2000). Ageing, tumour necrosis factor‐alpha (TNF‐alpha) and atherosclerosis. Clinical and Experimental Immunology, 121(2), 255–260. 10.1046/j.1365-2249.2000.01281.x 10931139 PMC1905691

[acel14008-bib-0016] Cahill, T. J. , Sun, X. , Ravaud, C. , Villa Del Campo, C. , Klaourakis, K. , Lupu, I. E. , Lord, A. M. , Browne, C. , Jacobsen, S. E. W. , Greaves, D. R. , Jackson, D. G. , Cowley, S. A. , James, W. , Choudhury, R. P. , Vieira, J. M. , & Riley, P. R. (2021). Tissue‐resident macrophages regulate lymphatic vessel growth and patterning in the developing heart. Development, 148(3), dev194563. 10.1242/dev.194563 33462113 PMC7875498

[acel14008-bib-0017] Cai, B. , Thorp, E. B. , Doran, A. C. , Sansbury, B. E. , Daemen, M. J. , Dorweiler, B. , Spite, M. , Fredman, G. , & Tabas, I. (2017). MerTK receptor cleavage promotes plaque necrosis and defective resolution in atherosclerosis. The Journal of Clinical Investigation, 127(2), 564–568. 10.1172/jci90520 28067670 PMC5272169

[acel14008-bib-0019] Chakarov, S. , Lim, H. Y. , Tan, L. , Lim, S. Y. , See, P. , Lum, J. , Zhang, X. M. , Foo, S. , Nakamizo, S. , Duan, K. , Kong, W. T. , Gentek, R. , Balachander, A. , Carbajo, D. , Bleriot, C. , Malleret, B. , Tam, J. K. C. , Baig, S. , Shabeer, M. , … Ginhoux, F. (2019). Two distinct interstitial macrophage populations coexist across tissues in specific subtissular niches. Science, 363(6432), eaau0964. 10.1126/science.aau0964 30872492

[acel14008-bib-0020] Chen, B. , & Frangogiannis, N. G. (2018). The role of macrophages in nonischemic heart failure. JACC Basic Transl Sci, 3(2), 245–248. 10.1016/j.jacbts.2018.03.001 30062210 PMC6060201

[acel14008-bib-0021] Chiao, Y. A. , Dai, Q. , Zhang, J. , Lin, J. , Lopez, E. F. , Ahuja, S. S. , Chou, Y. M. , Lindsey, M. L. , & Jin, Y. F. (2011). Multi‐analyte profiling reveals matrix metalloproteinase‐9 and monocyte chemotactic protein‐1 as plasma biomarkers of cardiac aging. Circulation. Cardiovascular Genetics, 4(4), 455–462. 10.1161/circgenetics.111.959981 21685172 PMC3158732

[acel14008-bib-0022] Cho, D. I. , Kim, M. R. , Jeong, H. Y. , Jeong, H. C. , Jeong, M. H. , Yoon, S. H. , Kim, Y. S. , & Ahn, Y. (2014). Mesenchymal stem cells reciprocally regulate the M1/M2 balance in mouse bone marrow‐derived macrophages. Experimental & Molecular Medicine, 46(1), e70. 10.1038/emm.2013.135 24406319 PMC3909888

[acel14008-bib-0023] Correia, L. C. , Lakatta, E. G. , O'Connor, F. C. , Becker, L. C. , Clulow, J. , Townsend, S. , Gerstenblith, G. , & Fleg, J. L. (2002). Attenuated cardiovascular reserve during prolonged submaximal cycle exercise in healthy older subjects. Journal of the American College of Cardiology, 40(7), 1290–1297. 10.1016/s0735-1097(02)02132-0 12383577

[acel14008-bib-0024] Dai, D. F. , & Rabinovitch, P. S. (2009). Cardiac aging in mice and humans: The role of mitochondrial oxidative stress. Trends in Cardiovascular Medicine, 19(7), 213–220. 10.1016/j.tcm.2009.12.004 20382344 PMC2858758

[acel14008-bib-0025] Dai, D. F. , Santana, L. F. , Vermulst, M. , Tomazela, D. M. , Emond, M. J. , MacCoss, M. J. , Gollahon, K. , Martin, G. M. , Loeb, L. A. , Ladiges, W. C. , & Rabinovitch, P. S. (2009). Overexpression of catalase targeted to mitochondria attenuates murine cardiac aging. Circulation, 119(21), 2789–2797. 10.1161/circulationaha.108.822403 19451351 PMC2858759

[acel14008-bib-0026] D'Amore, P. A. , & Alcaide, P. (2022). Macrophage efferocytosis with VEGFC and lymphangiogenesis: Rescuing the broken heart. The Journal of Clinical Investigation, 132(9), e158703. 10.1172/jci158703 35499075 PMC9057620

[acel14008-bib-0027] Davis, F. M. , & Gallagher, K. A. (2019). Epigenetic mechanisms in monocytes/macrophages regulate inflammation in Cardiometabolic and vascular disease. Arteriosclerosis, Thrombosis, and Vascular Biology, 39(4), 623–634. 10.1161/atvbaha.118.312135 30760015 PMC6438376

[acel14008-bib-0028] DeBerge, M. , Shah, S. J. , Wilsbacher, L. , & Thorp, E. B. (2019). Macrophages in heart failure with reduced versus preserved ejection fraction. Trends in Molecular Medicine, 25(4), 328–340. 10.1016/j.molmed.2019.01.002 30737012 PMC6552663

[acel14008-bib-0029] DeLeon‐Pennell, K. Y. , Meschiari, C. A. , Jung, M. , & Lindsey, M. L. (2017). Matrix Metalloproteinases in myocardial infarction and heart failure. Progress in Molecular Biology and Translational Science, 147, 75–100. 10.1016/bs.pmbts.2017.02.001 28413032 PMC5576003

[acel14008-bib-0030] Deniset, J. F. , Belke, D. , Lee, W. Y. , Jorch, S. K. , Deppermann, C. , Hassanabad, A. F. , Turnbull, J. D. , Teng, G. , Rozich, I. , Hudspeth, K. , Kanno, Y. , Brooks, S. R. , Hadjantonakis, A. K. , O'Shea, J. J. , Weber, G. F. , Fedak, P. W. M. , & Kubes, P. (2019). Gata6(+) pericardial cavity macrophages relocate to the injured heart and prevent cardiac fibrosis. Immunity, 51(1), 131–140.e135. 10.1016/j.immuni.2019.06.010 31315031 PMC7574643

[acel14008-bib-0031] Dick, S. A. , Macklin, J. A. , Nejat, S. , Momen, A. , Clemente‐Casares, X. , Althagafi, M. G. , Chen, J. , Kantores, C. , Hosseinzadeh, S. , Aronoff, L. , Wong, A. , Zaman, R. , Barbu, I. , Besla, R. , Lavine, K. J. , Razani, B. , Ginhoux, F. , Husain, M. , Cybulsky, M. I. , … Epelman, S. (2019). Self‐renewing resident cardiac macrophages limit adverse remodeling following myocardial infarction. Nature Immunology, 20(1), 29–39. 10.1038/s41590-018-0272-2 30538339 PMC6565365

[acel14008-bib-0032] Dodd, M. S. , Ball, D. R. , Schroeder, M. A. , Le Page, L. M. , Atherton, H. J. , Heather, L. C. , Seymour, A. M. , Ashrafian, H. , Watkins, H. , Clarke, K. , & Tyler, D. J. (2012). In vivo alterations in cardiac metabolism and function in the spontaneously hypertensive rat heart. Cardiovascular Research, 95(1), 69–76. 10.1093/cvr/cvs164 22593200 PMC4617603

[acel14008-bib-0033] Donato, A. J. , Machin, D. R. , & Lesniewski, L. A. (2018). Mechanisms of dysfunction in the aging vasculature and role in age‐related disease. Circulation Research, 123(7), 825–848. 10.1161/circresaha.118.312563 30355078 PMC6207260

[acel14008-bib-0034] Epelman, S. , Lavine, K. J. , Beaudin, A. E. , Sojka, D. K. , Carrero, J. A. , Calderon, B. , Brija, T. , Gautier, E. L. , Ivanov, S. , Satpathy, A. T. , Schilling, J. D. , Schwendener, R. , Sergin, I. , Razani, B. , Forsberg, E. C. , Yokoyama, W. M. , Unanue, E. R. , Colonna, M. , Randolph, G. J. , & Mann, D. L. (2014). Embryonic and adult‐derived resident cardiac macrophages are maintained through distinct mechanisms at steady state and during inflammation. Immunity, 40(1), 91–104. 10.1016/j.immuni.2013.11.019 24439267 PMC3923301

[acel14008-bib-0035] Fan, D. , Takawale, A. , Lee, J. , & Kassiri, Z. (2012). Cardiac fibroblasts, fibrosis and extracellular matrix remodeling in heart disease. Fibrogenesis & Tissue Repair, 5(1), 15. 10.1186/1755-1536-5-15 22943504 PMC3464725

[acel14008-bib-0036] Foreman, K. J. , Marquez, N. , Dolgert, A. , Fukutaki, K. , Fullman, N. , McGaughey, M. , Pletcher, M. A. , Smith, A. E. , Tang, K. , Yuan, C. W. , Brown, J. C. , Friedman, J. , He, J. , Heuton, K. R. , Holmberg, M. , Patel, D. J. , Reidy, P. , Carter, A. , Cercy, K. , … Murray, C. J. L. (2018). Forecasting life expectancy, years of life lost, and all‐cause and cause‐specific mortality for 250 causes of death: Reference and alternative scenarios for 2016‐40 for 195 countries and territories. Lancet, 392(10159), 2052–2090. 10.1016/s0140-6736(18)31694-5 30340847 PMC6227505

[acel14008-bib-0037] Franceschi, C. , Bonafè, M. , Valensin, S. , Olivieri, F. , De Luca, M. , Ottaviani, E. , & De Benedictis, G. (2000). Inflamm‐aging. An evolutionary perspective on immunosenescence. Annals of the New York Academy of Sciences, 908, 244–254. 10.1111/j.1749-6632.2000.tb06651.x 10911963

[acel14008-bib-0038] Franceschi, C. , & Campisi, J. (2014). Chronic inflammation (inflammaging) and its potential contribution to age‐associated diseases. The Journals of Gerontology. Series A, Biological Sciences and Medical Sciences, 69(Suppl 1), S4–S9. 10.1093/gerona/glu057 24833586

[acel14008-bib-0039] Francisco, V. , Pino, J. , Campos‐Cabaleiro, V. , Ruiz‐Fernández, C. , Mera, A. , Gonzalez‐Gay, M. A. , Gómez, R. , & Gualillo, O. (2018). Obesity, fat mass and immune system: Role for leptin. Frontiers in Physiology, 9, 640. 10.3389/fphys.2018.00640 29910742 PMC5992476

[acel14008-bib-0040] Frangogiannis, N. G. (2008). The immune system and cardiac repair. Pharmacological Research, 58(2), 88–111. 10.1016/j.phrs.2008.06.007 18620057 PMC2642482

[acel14008-bib-0041] Frangogiannis, N. G. (2012). Regulation of the inflammatory response in cardiac repair. Circulation Research, 110(1), 159–173. 10.1161/circresaha.111.243162 22223212 PMC3690135

[acel14008-bib-0042] Freeman, R. V. , & Otto, C. M. (2005). Spectrum of calcific aortic valve disease: Pathogenesis, disease progression, and treatment strategies. Circulation, 111(24), 3316–3326. 10.1161/circulationaha.104.486738 15967862

[acel14008-bib-0043] Galioto, A. , Dominguez, L. J. , Pineo, A. , Ferlisi, A. , Putignano, E. , Belvedere, M. , Costanza, G. , & Barbagallo, M. (2008). Cardiovascular risk factors in centenarians. Experimental Gerontology, 43(2), 106–113. 10.1016/j.exger.2007.06.009 17689040

[acel14008-bib-0044] Gautier, E. L. , Shay, T. , Miller, J. , Greter, M. , Jakubzick, C. , Ivanov, S. , Helft, J. , Chow, A. , Elpek, K. G. , Gordonov, S. , Mazloom, A. R. , Ma'ayan, A. , Chua, W. J. , Hansen, T. H. , Turley, S. J. , Merad, M. , & Randolph, G. J. (2012). Gene‐expression profiles and transcriptional regulatory pathways that underlie the identity and diversity of mouse tissue macrophages. Nature Immunology, 13(11), 1118–1128. 10.1038/ni.2419 23023392 PMC3558276

[acel14008-bib-0045] Ginhoux, F. , Greter, M. , Leboeuf, M. , Nandi, S. , See, P. , Gokhan, S. , Mehler, M. F. , Conway, S. J. , Ng, L. G. , Stanley, E. R. , Samokhvalov, I. M. , & Merad, M. (2010). Fate mapping analysis reveals that adult microglia derive from primitive macrophages. Science, 330(6005), 841–845. 10.1126/science.1194637 20966214 PMC3719181

[acel14008-bib-0046] Ginhoux, F. , & Jung, S. (2014). Monocytes and macrophages: Developmental pathways and tissue homeostasis. Nature Reviews. Immunology, 14(6), 392–404. 10.1038/nri3671 24854589

[acel14008-bib-0047] Gombozhapova, A. , Rogovskaya, Y. , Shurupov, V. , Rebenkova, M. , Kzhyshkowska, J. , Popov, S. V. , Karpov, R. S. , & Ryabov, V. (2017). Macrophage activation and polarization in post‐infarction cardiac remodeling. Journal of Biomedical Science, 24(1), 13. 10.1186/s12929-017-0322-3 28173864 PMC5297120

[acel14008-bib-0048] Gomez Perdiguero, E. , Klapproth, K. , Schulz, C. , Busch, K. , Azzoni, E. , Crozet, L. , Garner, H. , Trouillet, C. , de Bruijn, M. F. , Geissmann, F. , & Rodewald, H. R. (2015). Tissue‐resident macrophages originate from yolk‐sac‐derived erythro‐myeloid progenitors. Nature, 518(7540), 547–551. 10.1038/nature13989 25470051 PMC5997177

[acel14008-bib-0049] Goradel, N. H. , Hour, F. G. , Negahdari, B. , Malekshahi, Z. V. , Hashemzehi, M. , Masoudifar, A. , & Mirzaei, H. (2018). Stem cell therapy: A new therapeutic option for cardiovascular diseases. Journal of Cellular Biochemistry, 119(1), 95–104. 10.1002/jcb.26169 28543595

[acel14008-bib-0050] Gordon, S. (2016). Phagocytosis: The legacy of Metchnikoff. Cell, 166(5), 1065–1068. 10.1016/j.cell.2016.08.017 27565334

[acel14008-bib-0051] Groh, L. , Keating, S. T. , Joosten, L. A. B. , Netea, M. G. , & Riksen, N. P. (2018). Monocyte and macrophage immunometabolism in atherosclerosis. Seminars in Immunopathology, 40(2), 203–214. 10.1007/s00281-017-0656-7 28971272 PMC5809534

[acel14008-bib-0052] Gruzdeva, O. , Uchasova, E. , Dyleva, Y. , Borodkina, D. , Akbasheva, O. , Belik, E. , Karetnikova, V. , Brel, N. , Kokov, A. , Kashtalap, V. , & Barbarash, O. (2018). Relationships between epicardial adipose tissue thickness and adipo‐fibrokine indicator profiles post‐myocardial infarction. Cardiovascular Diabetology, 17(1), 40. 10.1186/s12933-018-0679-y 29548286 PMC5855976

[acel14008-bib-0053] Guilliams, M. , De Kleer, I. , Henri, S. , Post, S. , Vanhoutte, L. , De Prijck, S. , Deswarte, K. , Malissen, B. , Hammad, H. , & Lambrecht, B. N. (2013). Alveolar macrophages develop from fetal monocytes that differentiate into long‐lived cells in the first week of life via GM‐CSF. The Journal of Experimental Medicine, 210(10), 1977–1992. 10.1084/jem.20131199 24043763 PMC3782041

[acel14008-bib-0054] Guzzardi, M. A. , & Iozzo, P. (2019). Brain functional imaging in obese and diabetic patients. Acta Diabetologica, 56(2), 135–144. 10.1007/s00592-018-1185-0 29959509

[acel14008-bib-0055] Han, C. , Nie, Y. , Lian, H. , Liu, R. , He, F. , Huang, H. , & Hu, S. (2015). Acute inflammation stimulates a regenerative response in the neonatal mouse heart. Cell Research, 25(10), 1137–1151. 10.1038/cr.2015.110 26358185 PMC4650627

[acel14008-bib-0056] Hare, J. M. , Fishman, J. E. , Gerstenblith, G. , DiFede Velazquez, D. L. , Zambrano, J. P. , Suncion, V. Y. , Tracy, M. , Ghersin, E. , Johnston, P. V. , Brinker, J. A. , Breton, E. , Davis‐Sproul, J. , Schulman, I. H. , Byrnes, J. , Mendizabal, A. M. , Lowery, M. H. , Rouy, D. , Altman, P. , Wong Po Foo, C. , … Lardo, A. (2012). Comparison of allogeneic vs autologous bone marrow–derived mesenchymal stem cells delivered by transendocardial injection in patients with ischemic cardiomyopathy: The POSEIDON randomized trial. JAMA, 308(22), 2369–2379. 10.1001/jama.2012.25321 23117550 PMC4762261

[acel14008-bib-0057] Hart, D. N. , & Fabre, J. W. (1981). Demonstration and characterization of Ia‐positive dendritic cells in the interstitial connective tissues of rat heart and other tissues, but not brain. The Journal of Experimental Medicine, 154(2), 347–361. 10.1084/jem.154.2.347 6943285 PMC2186417

[acel14008-bib-0058] Heidt, T. , Courties, G. , Dutta, P. , Sager, H. B. , Sebas, M. , Iwamoto, Y. , Sun, Y. , Da Silva, N. , Panizzi, P. , van der Laan, A. M. , Swirski, F. K. , Weissleder, R. , & Nahrendorf, M. (2014). Differential contribution of monocytes to heart macrophages in steady‐state and after myocardial infarction. Circulation Research, 115(2), 284–295. 10.1161/circresaha.115.303567 24786973 PMC4082439

[acel14008-bib-0059] Hilgendorf, I. , Gerhardt, L. M. , Tan, T. C. , Winter, C. , Holderried, T. A. , Chousterman, B. G. , Iwamoto, Y. , Liao, R. , Zirlik, A. , Scherer‐Crosbie, M. , Hedrick, C. C. , Libby, P. , Nahrendorf, M. , Weissleder, R. , & Swirski, F. K. (2014). Ly‐6Chigh monocytes depend on Nr4a1 to balance both inflammatory and reparative phases in the infarcted myocardium. Circulation Research, 114(10), 1611–1622. 10.1161/circresaha.114.303204 24625784 PMC4017349

[acel14008-bib-0060] Horckmans, M. , Bianchini, M. , Santovito, D. , Megens, R. T. A. , Springael, J. Y. , Negri, I. , Vacca, M. , Di Eusanio, M. , Moschetta, A. , Weber, C. , Duchene, J. , & Steffens, S. (2018). Pericardial adipose tissue regulates Granulopoiesis, fibrosis, and cardiac function after myocardial infarction. Circulation, 137(9), 948–960. 10.1161/circulationaha.117.028833 29167227

[acel14008-bib-0061] Huang, C. K. , Dai, D. , Xie, H. , Zhu, Z. , Hu, J. , Su, M. , Liu, M. , Lu, L. , Shen, W. , Ning, G. , Wang, J. , Zhang, R. , & Yan, X. (2020). Lgr4 governs a pro‐inflammatory program in macrophages to antagonize Post‐infarction cardiac repair. Circulation Research, 127(8), 953–973. 10.1161/circresaha.119.315807 32600176

[acel14008-bib-0062] Hulsmans, M. , Clauss, S. , Xiao, L. , Aguirre, A. D. , King, K. R. , Hanley, A. , Hucker, W. J. , Wülfers, E. M. , Seemann, G. , Courties, G. , Iwamoto, Y. , Sun, Y. , Savol, A. J. , Sager, H. B. , Lavine, K. J. , Fishbein, G. A. , Capen, D. E. , Da Silva, N. , Miquerol, L. , … Nahrendorf, M. (2017). Macrophages facilitate electrical conduction in the heart. Cell, 169(3), 510–522. 10.1016/j.cell.2017.03.050 28431249 PMC5474950

[acel14008-bib-0063] Hulsmans, M. , Sager, H. B. , Roh, J. D. , Valero‐Muñoz, M. , Houstis, N. E. , Iwamoto, Y. , Sun, Y. , Wilson, R. M. , Wojtkiewicz, G. , Tricot, B. , Osborne, M. T. , Hung, J. , Vinegoni, C. , Naxerova, K. , Sosnovik, D. E. , Zile, M. R. , Bradshaw, A. D. , Liao, R. , Tawakol, A. , … Nahrendorf, M. (2018). Cardiac macrophages promote diastolic dysfunction. The Journal of Experimental Medicine, 215(2), 423–440. 10.1084/jem.20171274 29339450 PMC5789416

[acel14008-bib-0064] Huynh, M. L. , Fadok, V. A. , & Henson, P. M. (2002). Phosphatidylserine‐dependent ingestion of apoptotic cells promotes TGF‐beta1 secretion and the resolution of inflammation. The Journal of Clinical Investigation, 109(1), 41–50. 10.1172/jci11638 11781349 PMC150814

[acel14008-bib-0065] Jia, D. , Jiang, H. , Weng, X. , Wu, J. , Bai, P. , Yang, W. , Wang, Z. , Hu, K. , Sun, A. , & Ge, J. (2019). Interleukin‐35 promotes macrophage survival and improves wound healing after myocardial infarction in mice. Circulation Research, 124(9), 1323–1336. 10.1161/circresaha.118.314569 30832557

[acel14008-bib-0066] Jin, X. , Yao, T. , Zhou, Z. , Zhu, J. , Zhang, S. , Hu, W. , & Shen, C. (2015). Advanced glycation end products enhance macrophages polarization into M1 phenotype through activating RAGE/NF‐κB pathway. BioMed Research International, 2015, 1–12. 10.1155/2015/732450 PMC4465680 26114112

[acel14008-bib-0067] Kain, V. , Ingle, K. A. , Kachman, M. , Baum, H. , Shanmugam, G. , Rajasekaran, N. S. , Young, M. E. , & Halade, G. V. (2018). Excess ω‐6 fatty acids influx in aging drives metabolic dysregulation, electrocardiographic alterations, and low‐grade chronic inflammation. American Journal of Physiology. Heart and Circulatory Physiology, 314(2), H160–h169. 10.1152/ajpheart.00297.2017 28986357 PMC5867649

[acel14008-bib-0068] Kanter, J. E. , Kramer, F. , Barnhart, S. , Averill, M. M. , Vivekanandan‐Giri, A. , Vickery, T. , Li, L. O. , Becker, L. , Yuan, W. , Chait, A. , Braun, K. R. , Potter‐Perigo, S. , Sanda, S. , Wight, T. N. , Pennathur, S. , Serhan, C. N. , Heinecke, J. W. , Coleman, R. A. , & Bornfeldt, K. E. (2012). Diabetes promotes an inflammatory macrophage phenotype and atherosclerosis through acyl‐CoA synthetase 1. Proceedings of the National Academy of Sciences of the United States of America, 109(12), E715–E724. 10.1073/pnas.1111600109 22308341 PMC3311324

[acel14008-bib-0069] Karavidas, A. , Lazaros, G. , Tsiachris, D. , & Pyrgakis, V. (2010). Aging and the cardiovascular system. Hellenic Journal of Cardiology, 51(5), 421–427. 20876055

[acel14008-bib-0070] Khare, V. , Sodhi, A. , & Singh, S. M. (1996). Effect of aging on the tumoricidal functions of murine peritoneal macrophages. Natural Immunity, 15(6), 285–294. 9523280

[acel14008-bib-0071] Koenig, A. L. , Shchukina, I. , Amrute, J. , Andhey, P. S. , Zaitsev, K. , Lai, L. , Bajpai, G. , Bredemeyer, A. , Smith, G. , Jones, C. , Terrebonne, E. , Rentschler, S. L. , Artyomov, M. N. , & Lavine, K. J. (2022). Single‐cell transcriptomics reveals cell‐type‐specific diversification in human heart failure. Nature Cardiovascular Research, 1(3), 263–280. 10.1038/s44161-022-00028-6 PMC9364913 35959412

[acel14008-bib-0072] Kong, P. , Christia, P. , & Frangogiannis, N. G. (2014). The pathogenesis of cardiac fibrosis. Cellular and Molecular Life Sciences, 71(4), 549–574. 10.1007/s00018-013-1349-6 23649149 PMC3769482

[acel14008-bib-0073] Lafuse, W. P. , Wozniak, D. J. , & Rajaram, M. V. S. (2020). Role of cardiac macrophages on cardiac inflammation, fibrosis and tissue repair. Cells, 10(1), 51. 10.3390/cells10010051 33396359 PMC7824389

[acel14008-bib-0074] Lakatta, E. G. (2003). Arterial and cardiac aging: Major shareholders in cardiovascular disease enterprises: Part III: Cellular and molecular clues to heart and arterial aging. Circulation, 107(3), 490–497. 10.1161/01.cir.0000048894.99865.02 12551876

[acel14008-bib-0075] Lakatta, E. G. , & Levy, D. (2003a). Arterial and cardiac aging: Major shareholders in cardiovascular disease enterprises: Part I: Aging arteries: A "set up" for vascular disease. Circulation, 107(1), 139–146. 10.1161/01.cir.0000048892.83521.58 12515756

[acel14008-bib-0076] Lakatta, E. G. , & Levy, D. (2003b). Arterial and cardiac aging: Major shareholders in cardiovascular disease enterprises: Part II: The aging heart in health: Links to heart disease. Circulation, 107(2), 346–354. 10.1161/01.cir.0000048893.62841.f7 12538439

[acel14008-bib-0077] Lancaster, G. I. , Langley, K. G. , Berglund, N. A. , Kammoun, H. L. , Reibe, S. , Estevez, E. , Weir, J. , Mellett, N. A. , Pernes, G. , Conway, J. R. W. , Lee, M. K. S. , Timpson, P. , Murphy, A. J. , Masters, S. L. , Gerondakis, S. , Bartonicek, N. , Kaczorowski, D. C. , Dinger, M. E. , Meikle, P. J. , … Febbraio, M. A. (2018). Evidence that TLR4 is not a receptor for saturated fatty acids but mediates lipid‐induced inflammation by reprogramming macrophage metabolism. Cell Metabolism, 27(5), 1096–1110.e1095. 10.1016/j.cmet.2018.03.014 29681442

[acel14008-bib-0078] Lauterbach, M. A. , & Wunderlich, F. T. (2017). Macrophage function in obesity‐induced inflammation and insulin resistance. Pflügers Archiv, 469(3–4), 385–396. 10.1007/s00424-017-1955-5 28233125 PMC5362664

[acel14008-bib-0079] Lavine, K. J. , Epelman, S. , Uchida, K. , Weber, K. J. , Nichols, C. G. , Schilling, J. D. , Ornitz, D. M. , Randolph, G. J. , & Mann, D. L. (2014). Distinct macrophage lineages contribute to disparate patterns of cardiac recovery and remodeling in the neonatal and adult heart. Proceedings of the National Academy of Sciences of the United States of America, 111(45), 16029–16034. 10.1073/pnas.1406508111 25349429 PMC4234568

[acel14008-bib-0080] Leid, J. , Carrelha, J. , Boukarabila, H. , Epelman, S. , Jacobsen, S. E. , & Lavine, K. J. (2016). Primitive embryonic macrophages are required for coronary development and maturation. Circulation Research, 118(10), 1498–1511. 10.1161/circresaha.115.308270 27009605 PMC5567774

[acel14008-bib-0081] Leuschner, F. , Dutta, P. , Gorbatov, R. , Novobrantseva, T. I. , Donahoe, J. S. , Courties, G. , Lee, K. M. , Kim, J. I. , Markmann, J. F. , Marinelli, B. , Panizzi, P. , Lee, W. W. , Iwamoto, Y. , Milstein, S. , Epstein‐Barash, H. , Cantley, W. , Wong, J. , Cortez‐Retamozo, V. , Newton, A. , … Nahrendorf, M. (2011). Therapeutic siRNA silencing in inflammatory monocytes in mice. Nature Biotechnology, 29(11), 1005–1010. 10.1038/nbt.1989 PMC3212614 21983520

[acel14008-bib-0082] Lewis, A. J. M. , Miller, J. J. , Lau, A. Z. , Curtis, M. K. , Rider, O. J. , Choudhury, R. P. , Neubauer, S. , Cunningham, C. H. , Carr, C. A. , & Tyler, D. J. (2018). Noninvasive Immunometabolic cardiac inflammation imaging using hyperpolarized magnetic resonance. Circulation Research, 122(8), 1084–1093. 10.1161/circresaha.117.312535 29440071 PMC5908252

[acel14008-bib-0083] Li, Y. , Feng, J. , Song, S. , Li, H. , Yang, H. , Zhou, B. , Li, Y. , Yue, Z. , Lian, H. , Liu, L. , Hu, S. , & Nie, Y. (2020). gp130 controls Cardiomyocyte proliferation and heart regeneration. Circulation, 142(10), 967–982. 10.1161/circulationaha.119.044484 32600062

[acel14008-bib-0084] Lian, J. , Yue, Y. , Yu, W. , & Zhang, Y. (2020). Immunosenescence: A key player in cancer development. Journal of Hematology & Oncology, 13(1), 151. 10.1186/s13045-020-00986-z 33168037 PMC7653700

[acel14008-bib-0085] Liao, X. , Shen, Y. , Zhang, R. , Sugi, K. , Vasudevan, N. T. , Alaiti, M. A. , Sweet, D. R. , Zhou, L. , Qing, Y. , Gerson, S. L. , Fu, C. , Wynshaw‐Boris, A. , Hu, R. , Schwartz, M. A. , Fujioka, H. , Richardson, B. , Cameron, M. J. , Hayashi, H. , Stamler, J. S. , & Jain, M. K. (2018). Distinct roles of resident and nonresident macrophages in nonischemic cardiomyopathy. Proceedings of the National Academy of Sciences of the United States of America, 115(20), E4661–E4669. 10.1073/pnas.1720065115 29712858 PMC5960298

[acel14008-bib-0086] Lim, S. Y. , Cho, D. I. , Jeong, H. Y. , Kang, H. J. , Kim, M. R. , Cho, M. , Kim, Y. S. , & Ahn, Y. (2018). Adjuvant role of macrophages in stem cell‐induced cardiac repair in rats. Experimental & Molecular Medicine, 50(11), 1–10. 10.1038/s12276-018-0171-5 PMC6218450 30397194

[acel14008-bib-0087] Lin, J. , Lopez, E. F. , Jin, Y. , Van Remmen, H. , Bauch, T. , Han, H. C. , & Lindsey, M. L. (2008). Age‐related cardiac muscle sarcopenia: Combining experimental and mathematical modeling to identify mechanisms. Experimental Gerontology, 43(4), 296–306. 10.1016/j.exger.2007.12.005 18221848 PMC2323436

[acel14008-bib-0088] Lindsey, M. L. , Goshorn, D. K. , Squires, C. E. , Escobar, G. P. , Hendrick, J. W. , Mingoia, J. T. , Sweterlitsch, S. E. , & Spinale, F. G. (2005). Age‐dependent changes in myocardial matrix metalloproteinase/tissue inhibitor of metalloproteinase profiles and fibroblast function. Cardiovascular Research, 66(2), 410–419. 10.1016/j.cardiores.2004.11.029 15820210

[acel14008-bib-0089] Liu, C. F. , & Tang, W. H. W. (2019). Epigenetics in cardiac hypertrophy and heart failure. JACC Basic Transl Sci, 4(8), 976–993. 10.1016/j.jacbts.2019.05.011 31909304 PMC6938823

[acel14008-bib-0090] Liu, X. , Chen, J. , Zhang, B. , Liu, G. , Zhao, H. , & Hu, Q. (2019). KDM3A inhibition modulates macrophage polarization to aggravate post‐MI injuries and accelerates adverse ventricular remodeling via an IRF4 signaling pathway. Cellular Signalling, 64, 109415. 10.1016/j.cellsig.2019.109415 31513837

[acel14008-bib-0091] Ma, Y. , Chiao, Y. A. , Clark, R. , Flynn, E. R. , Yabluchanskiy, A. , Ghasemi, O. , Zouein, F. , Lindsey, M. L. , & Jin, Y. F. (2015). Deriving a cardiac ageing signature to reveal MMP‐9‐dependent inflammatory signalling in senescence. Cardiovascular Research, 106(3), 421–431. 10.1093/cvr/cvv128 25883218 PMC4498140

[acel14008-bib-0092] Ma, Y. , Chiao, Y. A. , Zhang, J. , Manicone, A. M. , Jin, Y. F. , & Lindsey, M. L. (2012). Matrix metalloproteinase‐28 deletion amplifies inflammatory and extracellular matrix responses to cardiac aging. Microscopy and Microanalysis, 18(1), 81–90. 10.1017/s1431927611012220 22153350 PMC3972008

[acel14008-bib-0093] Ma, Y. , Mouton, A. J. , & Lindsey, M. L. (2018). Cardiac macrophage biology in the steady‐state heart, the aging heart, and following myocardial infarction. Translational Research, 191, 15–28. 10.1016/j.trsl.2017.10.001 29106912 PMC5846093

[acel14008-bib-0094] Ma, Z. G. , Yuan, Y. P. , Wu, H. M. , Zhang, X. , & Tang, Q. Z. (2018). Cardiac fibrosis: New insights into the pathogenesis. International Journal of Biological Sciences, 14(12), 1645–1657. 10.7150/ijbs.28103 30416379 PMC6216032

[acel14008-bib-0095] Mahbub, S. , Deburghgraeve, C. R. , & Kovacs, E. J. (2012). Advanced age impairs macrophage polarization. Journal of Interferon & Cytokine Research, 32(1), 18–26. 10.1089/jir.2011.0058 22175541 PMC3255514

[acel14008-bib-0096] Martinez, F. O. , Sica, A. , Mantovani, A. , & Locati, M. (2008). Macrophage activation and polarization. Frontiers in Bioscience, 13, 453–461. 10.2741/2692 17981560

[acel14008-bib-0097] McDonagh, T. A. , Metra, M. , Adamo, M. , Gardner, R. S. , Baumbach, A. , Böhm, M. , Burri, H. , Butler, J. , Čelutkienė, J. , Chioncel, O. , Cleland, J. G. F. , Coats, A. J. S. , Crespo‐Leiro, M. G. , Farmakis, D. , Gilard, M. , Heymans, S. , Hoes, A. W. , Jaarsma, T. , Jankowska, E. A. , … Kathrine Skibelund, A. (2022). 2021 ESC guidelines for the diagnosis and treatment of acute and chronic heart failure: Developed by the task force for the diagnosis and treatment of acute and chronic heart failure of the European Society of Cardiology (ESC). With the special contribution of the heart failure association (HFA) of the ESC. European Journal of Heart Failure, 24(1), 4–131. 10.1002/ejhf.2333 35083827

[acel14008-bib-0098] Meschiari, C. A. , Ero, O. K. , Pan, H. , Finkel, T. , & Lindsey, M. L. (2017). The impact of aging on cardiac extracellular matrix. Geroscience, 39(1), 7–18. 10.1007/s11357-017-9959-9 28299638 PMC5352584

[acel14008-bib-0099] Meyer, I. S. , Goetzke, C. C. , Kespohl, M. , Sauter, M. , Heuser, A. , Eckstein, V. , Vornlocher, H. P. , Anderson, D. G. , Haas, J. , Meder, B. , Katus, H. A. , Klingel, K. , Beling, A. , & Leuschner, F. (2018). Silencing the CSF‐1 Axis using nanoparticle encapsulated siRNA mitigates viral and autoimmune myocarditis. Frontiers in Immunology, 9, 2303. 10.3389/fimmu.2018.02303 30349538 PMC6186826

[acel14008-bib-0100] Mia, M. M. , Cibi, D. M. , Abdul Ghani, S. A. B. , Song, W. , Tee, N. , Ghosh, S. , Mao, J. , Olson, E. N. , & Singh, M. K. (2020). YAP/TAZ deficiency reprograms macrophage phenotype and improves infarct healing and cardiac function after myocardial infarction. PLoS Biology, 18(12), e3000941. 10.1371/journal.pbio.3000941 33264286 PMC7735680

[acel14008-bib-0101] Mishra, P. K. , Ying, W. , Nandi, S. S. , Bandyopadhyay, G. K. , Patel, K. K. , & Mahata, S. K. (2017). Diabetic cardiomyopathy: An Immunometabolic perspective. Front Endocrinol (Lausanne), 8, 72. 10.3389/fendo.2017.00072 28439258 PMC5384479

[acel14008-bib-0102] Molawi, K. , Wolf, Y. , Kandalla, P. K. , Favret, J. , Hagemeyer, N. , Frenzel, K. , Pinto, A. R. , Klapproth, K. , Henri, S. , Malissen, B. , Rodewald, H. R. , Rosenthal, N. A. , Bajenoff, M. , Prinz, M. , Jung, S. , & Sieweke, M. H. (2014). Progressive replacement of embryo‐derived cardiac macrophages with age. The Journal of Experimental Medicine, 211(11), 2151–2158. 10.1084/jem.20140639 25245760 PMC4203946

[acel14008-bib-0103] Mosser, D. M. , & Edwards, J. P. (2008). Exploring the full spectrum of macrophage activation. Nature Reviews. Immunology, 8(12), 958–969. 10.1038/nri2448 PMC2724991 19029990

[acel14008-bib-0104] Mouton, A. J. , DeLeon‐Pennell, K. Y. , Rivera Gonzalez, O. J. , Flynn, E. R. , Freeman, T. C. , Saucerman, J. J. , Garrett, M. R. , Ma, Y. , Harmancey, R. , & Lindsey, M. L. (2018). Mapping macrophage polarization over the myocardial infarction time continuum. Basic Research in Cardiology, 113(4), 26. 10.1007/s00395-018-0686-x 29868933 PMC5986831

[acel14008-bib-0105] Mouton, A. J. , Li, X. , Hall, M. E. , & Hall, J. E. (2020). Obesity, hypertension, and cardiac dysfunction: Novel roles of Immunometabolism in macrophage activation and inflammation. Circulation Research, 126(6), 789–806. 10.1161/circresaha.119.312321 32163341 PMC7255054

[acel14008-bib-0106] Mouton, A. J. , Ma, Y. , Rivera Gonzalez, O. J. , Daseke, M. J., 2nd , Flynn, E. R. , Freeman, T. C. , Garrett, M. R. , DeLeon‐Pennell, K. Y. , & Lindsey, M. L. (2019). Fibroblast polarization over the myocardial infarction time continuum shifts roles from inflammation to angiogenesis. Basic Research in Cardiology, 114(2), 6. 10.1007/s00395-019-0715-4 30635789 PMC6329742

[acel14008-bib-0107] Murphy, A. M. , Wong, A. L. , & Bezuhly, M. (2015). Modulation of angiotensin II signaling in the prevention of fibrosis. Fibrogenesis & Tissue Repair, 8, 7. 10.1186/s13069-015-0023-z 25949522 PMC4422447

[acel14008-bib-0108] Nahrendorf, M. , & Swirski, F. K. (2016). Innate immune cells in ischaemic heart disease: Does myocardial infarction beget myocardial infarction? European Heart Journal, 37(11), 868–872. 10.1093/eurheartj/ehv453 26351395 PMC4789592

[acel14008-bib-0109] Nahrendorf, M. , Swirski, F. K. , Aikawa, E. , Stangenberg, L. , Wurdinger, T. , Figueiredo, J. L. , Libby, P. , Weissleder, R. , & Pittet, M. J. (2007). The healing myocardium sequentially mobilizes two monocyte subsets with divergent and complementary functions. The Journal of Experimental Medicine, 204(12), 3037–3047. 10.1084/jem.20070885 18025128 PMC2118517

[acel14008-bib-0110] Namgaladze, D. , & Brüne, B. (2016). Macrophage fatty acid oxidation and its roles in macrophage polarization and fatty acid‐induced inflammation. Biochimica et Biophysica Acta, 1861(11), 1796–1807. 10.1016/j.bbalip.2016.09.002 27614008

[acel14008-bib-0111] Nassimiha, D. , Aronow, W. S. , Ahn, C. , & Goldman, M. E. (2001). Association of coronary risk factors with progression of valvular aortic stenosis in older persons. The American Journal of Cardiology, 87(11), 1313–1314. 10.1016/s0002-9149(01)01531-4 11377366

[acel14008-bib-0112] Nicolás‐Ávila, J. A. , Lechuga‐Vieco, A. V. , Esteban‐Martínez, L. , Sánchez‐Díaz, M. , Díaz‐García, E. , Santiago, D. J. , Rubio‐Ponce, A. , Li, J. L. , Balachander, A. , Quintana, J. A. , Martínez‐de‐Mena, R. , Ibáñez, B. , Torres, M. , Priori, S. G. , Bueno, H. , Vázquez, J. , Cordero, M. D. , Bernal, J. A. , Enríquez, J. A. , … Hidalgo, A. (2020). A network of macrophages supports mitochondrial homeostasis in the heart. Cell, 183(1), 94–109.e123. 10.1016/j.cell.2020.08.031 32937105

[acel14008-bib-0113] Olsen, M. H. , Wachtell, K. , Bella, J. N. , Gerdts, E. , Palmieri, V. , Nieminen, M. S. , Smith, G. , Ibsen, H. , & Devereux, R. B. (2005). Aortic valve sclerosis relates to cardiovascular events in patients with hypertension (a LIFE substudy). The American Journal of Cardiology, 95(1), 132–136. 10.1016/j.amjcard.2004.08.080 15619412

[acel14008-bib-0114] Otto, C. M. , Lind, B. K. , Kitzman, D. W. , Gersh, B. J. , & Siscovick, D. S. (1999). Association of aortic‐valve sclerosis with cardiovascular mortality and morbidity in the elderly. The New England Journal of Medicine, 341(3), 142–147. 10.1056/nejm199907153410302 10403851

[acel14008-bib-0115] Pardali, E. , Dimmeler, S. , Zeiher, A. M. , & Rieger, M. A. (2020). Clonal hematopoiesis, aging, and cardiovascular diseases. Experimental Hematology, 83, 95–104. 10.1016/j.exphem.2019.12.006 31891750

[acel14008-bib-0116] Park, D. , Han, C. Z. , Elliott, M. R. , Kinchen, J. M. , Trampont, P. C. , Das, S. , Collins, S. , Lysiak, J. J. , Hoehn, K. L. , & Ravichandran, K. S. (2011). Continued clearance of apoptotic cells critically depends on the phagocyte Ucp2 protein. Nature, 477(7363), 220–224. 10.1038/nature10340 21857682 PMC3513690

[acel14008-bib-0117] Pavlou, S. , Lindsay, J. , Ingram, R. , Xu, H. , & Chen, M. (2018). Sustained high glucose exposure sensitizes macrophage responses to cytokine stimuli but reduces their phagocytic activity. BMC Immunology, 19(1), 24. 10.1186/s12865-018-0261-0 29996768 PMC6042333

[acel14008-bib-0118] Pelegrin, P. , & Surprenant, A. (2009). Dynamics of macrophage polarization reveal new mechanism to inhibit IL‐1beta release through pyrophosphates. The EMBO Journal, 28(14), 2114–2127. 10.1038/emboj.2009.163 19536133 PMC2699392

[acel14008-bib-0119] Perls, T. , & Terry, D. (2003). Understanding the determinants of exceptional longevity. Annals of Internal Medicine, 139(5 Pt 2), 445–449. 10.7326/0003-4819-139-5_part_2-200309021-00013 12965974

[acel14008-bib-0120] Pinto, A. R. , Ilinykh, A. , Ivey, M. J. , Kuwabara, J. T. , D'Antoni, M. L. , Debuque, R. , Chandran, A. , Wang, L. , Arora, K. , Rosenthal, N. A. , & Tallquist, M. D. (2016). Revisiting cardiac cellular composition. Circulation Research, 118(3), 400–409. 10.1161/circresaha.115.307778 26635390 PMC4744092

[acel14008-bib-0121] Ramos, G. , Hofmann, U. , & Frantz, S. (2016). Myocardial fibrosis seen through the lenses of T‐cell biology. Journal of Molecular and Cellular Cardiology, 92, 41–45. 10.1016/j.yjmcc.2016.01.018 26804387

[acel14008-bib-0122] Ramos, G. C. , van den Berg, A. , Nunes‐Silva, V. , Weirather, J. , Peters, L. , Burkard, M. , Friedrich, M. , Pinnecker, J. , Abeßer, M. , Heinze, K. G. , Schuh, K. , Beyersdorf, N. , Kerkau, T. , Demengeot, J. , Frantz, S. , & Hofmann, U. (2017). Myocardial aging as a T‐cell‐mediated phenomenon. Proceedings of the National Academy of Sciences of the United States of America, 114(12), E2420–e2429. 10.1073/pnas.1621047114 28255084 PMC5373357

[acel14008-bib-0123] Ren, J. , & Zhang, Y. (2018). Targeting autophagy in aging and aging‐related cardiovascular diseases. Trends in Pharmacological Sciences, 39(12), 1064–1076. 10.1016/j.tips.2018.10.005 30458935 PMC6251315

[acel14008-bib-0124] Renshaw, M. , Rockwell, J. , Engleman, C. , Gewirtz, A. , Katz, J. , & Sambhara, S. (2002). Cutting edge: Impaired toll‐like receptor expression and function in aging. Journal of Immunology, 169(9), 4697–4701. 10.4049/jimmunol.169.9.4697 12391175

[acel14008-bib-0125] Revelo, X. S. , Parthiban, P. , Chen, C. , Barrow, F. , Fredrickson, G. , Wang, H. , Yücel, D. , Herman, A. , & van Berlo, J. H. (2021). Cardiac resident macrophages prevent fibrosis and stimulate angiogenesis. Circulation Research, 129(12), 1086–1101. 10.1161/circresaha.121.319737 34645281 PMC8638822

[acel14008-bib-0126] Satpathy, A. T. , Kc, W. , Albring, J. C. , Edelson, B. T. , Kretzer, N. M. , Bhattacharya, D. , Murphy, T. L. , & Murphy, K. M. (2012). Zbtb46 expression distinguishes classical dendritic cells and their committed progenitors from other immune lineages. The Journal of Experimental Medicine, 209(6), 1135–1152. 10.1084/jem.20120030 22615127 PMC3371733

[acel14008-bib-0127] Savoia, C. , Sada, L. , Zezza, L. , Pucci, L. , Lauri, F. M. , Befani, A. , Alonzo, A. , & Volpe, M. (2011). Vascular inflammation and endothelial dysfunction in experimental hypertension. International Journal of Hypertension, 2011, 281240. 10.4061/2011/281240 21915370 PMC3170891

[acel14008-bib-0128] Selim, A. J. , Fincke, G. , Berlowitz, D. R. , Miller, D. R. , Qian, S. X. , Lee, A. , Cong, Z. , Rogers, W. , Selim, B. J. , Ren, X. S. , Spiro, A., III , & Kazis, L. E. (2005). Comprehensive health status assessment of centenarians: Results from the 1999 large health survey of veteran enrollees. The Journals of Gerontology. Series A, Biological Sciences and Medical Sciences, 60(4), 515–519. 10.1093/gerona/60.4.515 15933394

[acel14008-bib-0129] Serhan, C. N. , & Savill, J. (2005). Resolution of inflammation: The beginning programs the end. Nature Immunology, 6(12), 1191–1197. 10.1038/ni1276 16369558

[acel14008-bib-0130] Shigeta, A. , Huang, V. , Zuo, J. , Besada, R. , Nakashima, Y. , Lu, Y. , Ding, Y. , Pellegrini, M. , Kulkarni, R. P. , Hsiai, T. , Deb, A. , Zhou, B. , Nakano, H. , & Nakano, A. (2019). Endocardially derived macrophages are essential for Valvular remodeling. Developmental Cell, 48(5), 617–630.e613. 10.1016/j.devcel.2019.01.021 30799229 PMC6440481

[acel14008-bib-0131] Simões, F. C. , & Riley, P. R. (2018). The ontogeny, activation and function of the epicardium during heart development and regeneration. Development, 145(7), dev155994. 10.1242/dev.155994 29592950

[acel14008-bib-0132] Soehnlein, O. , & Lindbom, L. (2010). Phagocyte partnership during the onset and resolution of inflammation. Nature Reviews. Immunology, 10(6), 427–439. 10.1038/nri2779 20498669

[acel14008-bib-0133] Spurthi, K. M. , Sarikhani, M. , Mishra, S. , Desingu, P. A. , Yadav, S. , Rao, S. , Maity, S. , Tamta, A. K. , Kumar, S. , Majumdar, S. , Jain, A. , Raghuraman, A. , Khan, D. , Singh, I. , Samuel, R. J. , Ramachandra, S. G. , Nandi, D. , & Sundaresan, N. R. (2018). Toll‐like receptor 2 deficiency hyperactivates the FoxO1 transcription factor and induces aging‐associated cardiac dysfunction in mice. The Journal of Biological Chemistry, 293(34), 13073–13089. 10.1074/jbc.RA118.001880 29929978 PMC6109936

[acel14008-bib-0134] Stevens, S. M. , von Gise, A. , VanDusen, N. , Zhou, B. , & Pu, W. T. (2016). Epicardium is required for cardiac seeding by yolk sac macrophages, precursors of resident macrophages of the adult heart. Developmental Biology, 413(2), 153–159. 10.1016/j.ydbio.2016.03.014 26988120 PMC5064848

[acel14008-bib-0135] Stewart, B. F. , Siscovick, D. , Lind, B. K. , Gardin, J. M. , Gottdiener, J. S. , Smith, V. E. , Kitzman, D. W. , & Otto, C. M. (1997). Clinical factors associated with calcific aortic valve disease. Cardiovascular health study. Journal of the American College of Cardiology, 29(3), 630–634. 10.1016/s0735-1097(96)00563-3 9060903

[acel14008-bib-0136] Stoppe, C. , Averdunk, L. , Goetzenich, A. , Soppert, J. , Marlier, A. , Kraemer, S. , Vieten, J. , Coburn, M. , Kowark, A. , Kim, B. S. , Marx, G. , Rex, S. , Ochi, A. , Leng, L. , Moeckel, G. , Linkermann, A. , El Bounkari, O. , Zarbock, A. , Bernhagen, J. , … Boor, P. (2018). The protective role of macrophage migration inhibitory factor in acute kidney injury after cardiac surgery. Science Translational Medicine, 10(441), eaan4886. 10.1126/scitranslmed.aan4886 29769287

[acel14008-bib-0137] Tannahill, G. M. , Curtis, A. M. , Adamik, J. , Palsson‐McDermott, E. M. , McGettrick, A. F. , Goel, G. , Frezza, C. , Bernard, N. J. , Kelly, B. , Foley, N. H. , Zheng, L. , Gardet, A. , Tong, Z. , Jany, S. S. , Corr, S. C. , Haneklaus, M. , Caffrey, B. E. , Pierce, K. , Walmsley, S. , … O'Neill, L. A. (2013). Succinate is an inflammatory signal that induces IL‐1β through HIF‐1α. Nature, 496(7444), 238–242. 10.1038/nature11986 23535595 PMC4031686

[acel14008-bib-0138] Toba, H. , Cannon, P. L. , Yabluchanskiy, A. , Iyer, R. P. , D'Armiento, J. , & Lindsey, M. L. (2017). Transgenic overexpression of macrophage matrix metalloproteinase‐9 exacerbates age‐related cardiac hypertrophy, vessel rarefaction, inflammation, and fibrosis. American Journal of Physiology. Heart and Circulatory Physiology, 312(3), H375–h383. 10.1152/ajpheart.00633.2016 28011588 PMC5402013

[acel14008-bib-0139] Tucker, N. R. , Chaffin, M. , Fleming, S. J. , Hall, A. W. , Parsons, V. A. , Bedi, K. C., Jr. , Akkad, A. D. , Herndon, C. N. , Arduini, A. , Papangeli, I. , Roselli, C. , Aguet, F. , Choi, S. H. , Ardlie, K. G. , Babadi, M. , Margulies, K. B. , Stegmann, C. M. , & Ellinor, P. T. (2020). Transcriptional and cellular diversity of the human heart. Circulation, 142(5), 466–482. 10.1161/circulationaha.119.045401 32403949 PMC7666104

[acel14008-bib-0140] Vagnozzi, R. J. , Maillet, M. , Sargent, M. A. , Khalil, H. , Johansen, A. K. Z. , Schwanekamp, J. A. , York, A. J. , Huang, V. , Nahrendorf, M. , Sadayappan, S. , & Molkentin, J. D. (2020). An acute immune response underlies the benefit of cardiac stem cell therapy. Nature, 577(7790), 405–409. 10.1038/s41586-019-1802-2 31775156 PMC6962570

[acel14008-bib-0141] van Furth, R. , & Cohn, Z. A. (1968). The origin and kinetics of mononuclear phagocytes. The Journal of Experimental Medicine, 128(3), 415–435. 10.1084/jem.128.3.415 5666958 PMC2138527

[acel14008-bib-0142] Vianello, E. , Marrocco‐Trischitta Massimiliano, M. , Dozio, E. , Bandera, F. , Tacchini, L. , Canciani, E. , Dellavia, C. , Schmitz, G. , Lorenzo, M. , & Massimiliano, C. R. (2019). Correlational study on altered epicardial adipose tissue as a stratification risk factor for valve disease progression through IL‐13 signaling. Journal of Molecular and Cellular Cardiology, 132, 210–218. 10.1016/j.yjmcc.2019.05.012 31102584

[acel14008-bib-0143] Wagner, J. U. G. , & Dimmeler, S. (2020). Cellular cross‐talks in the diseased and aging heart. Journal of Molecular and Cellular Cardiology, 138, 136–146. 10.1016/j.yjmcc.2019.11.152 31783034

[acel14008-bib-0144] Wan, E. , Yeap, X. Y. , Dehn, S. , Terry, R. , Novak, M. , Zhang, S. , Iwata, S. , Han, X. , Homma, S. , Drosatos, K. , Lomasney, J. , Engman, D. M. , Miller, S. D. , Vaughan, D. E. , Morrow, J. P. , Kishore, R. , & Thorp, E. B. (2013). Enhanced efferocytosis of apoptotic cardiomyocytes through myeloid‐epithelial‐reproductive tyrosine kinase links acute inflammation resolution to cardiac repair after infarction. Circulation Research, 113(8), 1004–1012. 10.1161/circresaha.113.301198 23836795 PMC3840464

[acel14008-bib-0145] Wang, M. , & Shah, A. M. (2015). Age‐associated pro‐inflammatory remodeling and functional phenotype in the heart and large arteries. Journal of Molecular and Cellular Cardiology, 83, 101–111. 10.1016/j.yjmcc.2015.02.004 25665458 PMC4459900

[acel14008-bib-0146] Wang, Y. , Li, C. , Zhao, R. , Qiu, Z. , Shen, C. , Wang, Z. , Liu, W. , Zhang, W. , Ge, J. , & Shi, B. (2021). CircUbe3a from M2 macrophage‐derived small extracellular vesicles mediates myocardial fibrosis after acute myocardial infarction. Theranostics, 11(13), 6315–6333. 10.7150/thno.52843 33995660 PMC8120198

[acel14008-bib-0147] Wang, Y. , Sano, S. , Yura, Y. , Ke, Z. , Sano, M. , Oshima, K. , Ogawa, H. , Horitani, K. , Min, K. D. , Miura‐Yura, E. , Kour, A. , Evans, M. A. , Zuriaga, M. A. , Hirschi, K. K. , Fuster, J. J. , Pietras, E. M. , & Walsh, K. (2020). Tet2‐mediated clonal hematopoiesis in nonconditioned mice accelerates age‐associated cardiac dysfunction. JCI Insight, 5(6), 135204. 10.1172/jci.insight.135204 32154790 PMC7213793

[acel14008-bib-0148] Wang, Z. , Cui, M. , Shah, A. M. , Ye, W. , Tan, W. , Min, Y. L. , Botten, G. A. , Shelton, J. M. , Liu, N. , Bassel‐Duby, R. , & Olson, E. N. (2019). Mechanistic basis of neonatal heart regeneration revealed by transcriptome and histone modification profiling. Proceedings of the National Academy of Sciences of the United States of America, 116(37), 18455–18465. 10.1073/pnas.1905824116 31451669 PMC6744882

[acel14008-bib-0149] Watanabe, R. , Hilhorst, M. , Zhang, H. , Zeisbrich, M. , Berry, G. J. , Wallis, B. B. , Harrison, D. G. , Giacomini, J. C. , Goronzy, J. J. , & Weyand, C. M. (2018). Glucose metabolism controls disease‐specific signatures of macrophage effector functions. JCI Insight, 3(20), e123047. 10.1172/jci.insight.123047 30333306 PMC6237479

[acel14008-bib-0150] Weber, K. T. , Sun, Y. , Bhattacharya, S. K. , Ahokas, R. A. , & Gerling, I. C. (2013). Myofibroblast‐mediated mechanisms of pathological remodelling of the heart. Nature Reviews. Cardiology, 10(1), 15–26. 10.1038/nrcardio.2012.158 23207731

[acel14008-bib-0151] Wei, X. , Zou, S. , Xie, Z. , Wang, Z. , Huang, N. , Cen, Z. , Hao Y , Zhang C , Chen Z , Zhao F , Hu Z , Teng X , Gui Y , Liu X , Zheng H , Zhou H , Chen S , Cheng J , Zeng F , … Li, J. (2022). EDIL3 deficiency ameliorates adverse cardiac remodelling by neutrophil extracellular traps (NET)‐mediated macrophage polarization. Cardiovascular Research, 118(9), 2179–2195. 34375400 10.1093/cvr/cvab269

[acel14008-bib-0152] Wicks, K. , Torbica, T. , & Mace, K. A. (2014). Myeloid cell dysfunction and the pathogenesis of the diabetic chronic wound. Seminars in Immunology, 26(4), 341–353. 10.1016/j.smim.2014.04.006 24954378

[acel14008-bib-0153] Wong, N. R. , Mohan, J. , Kopecky, B. J. , Guo, S. , Du, L. , Leid, J. , Feng, G. , Lokshina, I. , Dmytrenko, O. , Luehmann, H. , Bajpai, G. , Ewald, L. , Bell, L. , Patel, N. , Bredemeyer, A. , Weinheimer, C. J. , Nigro, J. M. , Kovacs, A. , Morimoto, S. , … Lavine, K. J. (2021). Resident cardiac macrophages mediate adaptive myocardial remodeling. Immunity, 54(9), 2072–2088.e2077. 10.1016/j.immuni.2021.07.003 34320366 PMC8446343

[acel14008-bib-0154] Wu, H. M. , Zhang, L. F. , Ding, P. S. , Liu, Y. J. , Wu, X. , & Zhou, J. N. (2014). Microglial activation mediates host neuronal survival induced by neural stem cells. Journal of Cellular and Molecular Medicine, 18(7), 1300–1312. 10.1111/jcmm.12281 24725889 PMC4124015

[acel14008-bib-0155] Xia, S. , Zhang, X. , Zheng, S. , Khanabdali, R. , Kalionis, B. , Wu, J. , Wan, W. , & Tai, X. (2016). An update on Inflamm‐aging: Mechanisms, prevention, and treatment. Journal of Immunology Research, 2016, 8426874. 10.1155/2016/8426874 27493973 PMC4963991

[acel14008-bib-0156] Xin, M. , Kim, Y. , Sutherland, L. B. , Murakami, M. , Qi, X. , McAnally, J. , Porrello, E. R. , Mahmoud, A. I. , Tan, W. , Shelton, J. M. , Richardson, J. A. , Sadek, H. A. , Bassel‐Duby, R. , & Olson, E. N. (2013). Hippo pathway effector yap promotes cardiac regeneration. Proceedings of the National Academy of Sciences of the United States of America, 110(34), 13839–13844. 10.1073/pnas.1313192110 23918388 PMC3752208

[acel14008-bib-0157] Yabluchanskiy, A. , Ma, Y. , Chiao, Y. A. , Lopez, E. F. , Voorhees, A. P. , Toba, H. , Hall, M. E. , Han, H. C. , Lindsey, M. L. , & Jin, Y. F. (2014). Cardiac aging is initiated by matrix metalloproteinase‐9‐mediated endothelial dysfunction. American Journal of Physiology. Heart and Circulatory Physiology, 306(10), H1398–H1407. 10.1152/ajpheart.00090.2014 24658018 PMC4024719

[acel14008-bib-0158] Yona, S. , Kim, K. W. , Wolf, Y. , Mildner, A. , Varol, D. , Breker, M. , Strauss‐Ayali, D. , Viukov, S. , Guilliams, M. , Misharin, A. , Hume, D. A. , Perlman, H. , Malissen, B. , Zelzer, E. , & Jung, S. (2013). Fate mapping reveals origins and dynamics of monocytes and tissue macrophages under homeostasis. Immunity, 38(1), 79–91. 10.1016/j.immuni.2012.12.001 23273845 PMC3908543

[acel14008-bib-0159] Zagórska, A. , Través, P. G. , Lew, E. D. , Dransfield, I. , & Lemke, G. (2014). Diversification of TAM receptor tyrosine kinase function. Nature Immunology, 15(10), 920–928. 10.1038/ni.2986 25194421 PMC4169336

[acel14008-bib-0160] Zaman, R. , Hamidzada, H. , Kantores, C. , Wong, A. , Dick, S. A. , Wang, Y. , Momen, A. , Aronoff, L. , Lin, J. , Razani, B. , Mital, S. , Billia, F. , Lavine, K. J. , Nejat, S. , & Epelman, S. (2021). Selective loss of resident macrophage‐derived insulin‐like growth factor‐1 abolishes adaptive cardiac growth to stress. Immunity, 54(9), 2057–2071.e2056. 10.1016/j.immuni.2021.07.006 34363749 PMC12797153

[acel14008-bib-0161] Zhang, B. , Xu, D. , She, L. , Wang, Z. , Yang, N. , Sun, R. , Zhang, Y. , Yan, C. , Wei, Q. , Aa, J. , Liu, B. , Wang, G. , & Xie, Y. (2018). Silybin inhibits NLRP3 inflammasome assembly through the NAD(+)/SIRT2 pathway in mice with nonalcoholic fatty liver disease. The FASEB Journal, 32(2), 757–767. 10.1096/fj.201700602R 28970254

[acel14008-bib-0162] Zhang, Q. , Raoof, M. , Chen, Y. , Sumi, Y. , Sursal, T. , Junger, W. , Brohi, K. , Itagaki, K. , & Hauser, C. J. (2010). Circulating mitochondrial DAMPs cause inflammatory responses to injury. Nature, 464(7285), 104–107. 10.1038/nature08780 20203610 PMC2843437

[acel14008-bib-0163] Zhang, S. , Weinberg, S. , DeBerge, M. , Gainullina, A. , Schipma, M. , Kinchen, J. M. , Ben‐Sahra, I. , Gius, D. R. , Yvan‐Charvet, L. , Chandel, N. S. , Schumacker, P. T. , & Thorp, E. B. (2019). Efferocytosis fuels requirements of fatty acid oxidation and the electron transport chain to polarize macrophages for tissue repair. Cell Metabolism, 29(2), 443–456.e445. 10.1016/j.cmet.2018.12.004 30595481 PMC6471613

